# Early aberrant DNA methylation events in a mouse model of acute myeloid leukemia

**DOI:** 10.1186/gm551

**Published:** 2014-04-30

**Authors:** Miriam Sonnet, Rainer Claus, Natalia Becker, Manuela Zucknick, Jana Petersen, Daniel B Lipka, Christopher C Oakes, Mindaugas Andrulis, Amelie Lier, Michael D Milsom, Tania Witte, Lei Gu, Soo-Zin Kim-Wanner, Peter Schirmacher, Michael Wulfert, Norbert Gattermann, Michael Lübbert, Frank Rosenbauer, Michael Rehli, Lars Bullinger, Dieter Weichenhan, Christoph Plass

**Affiliations:** 1 Department of Epigenomics and Cancer Risk Factors, German Cancer Research Center (DKFZ), D-69120 Heidelberg, Germany; 2 Division of Biostatistics, German Cancer Research Center (DKFZ), D-69120 Heidelberg, Germany; 3 Department of General Pathology, Institute of Pathology, University Heidelberg, D-69120 Heidelberg, Germany; 4 Division of Stem Cells and Cancer, German Cancer Research Center (DKFZ), D-69120 Heidelberg, Germany; 5 Department of Hematology, Oncology and Clinical Immunology, Heinrich-Heine University, D-40225 Düsseldorf, Germany; 6 Department of Hematology/Oncology, University Medical Center, D-79106 Freiburg, Germany; 7 Institute of Molecular Tumor Biology, Westfälische Wilhelms Universität, D-48149 Münster, Germany; 8 Department of Hematology and Oncology, University Hospital Regensburg, D-93042 Regensburg, Germany; 9 Department of Internal Medicine III, University of Ulm, D-89081 Ulm, Germany; 10 Division of Theoretical Bioinformatics, German Cancer Research Center (DKFZ), D-69120 Heidelberg, Germany

## Abstract

**Background:**

Aberrant DNA methylation is frequently found in human malignancies including acute myeloid leukemia (AML). While most studies focus on later disease stages, the onset of aberrant DNA methylation events and their dynamics during leukemic progression are largely unknown.

**Methods:**

We screened genome-wide for aberrant CpG island methylation in three disease stages of a murine AML model that is driven by hypomorphic expression of the hematopoietic transcription factor PU.1. DNA methylation levels of selected genes were correlated with methylation levels of CD34+ cells and lineage negative, CD127-, c-Kit+, Sca-1+ cells; common myeloid progenitors; granulocyte-macrophage progenitors; and megakaryocyte-erythroid progenitors.

**Results:**

We identified 1,184 hypermethylated array probes covering 762 associated genes in the preleukemic stage. During disease progression, the number of hypermethylated genes increased to 5,465 in the late leukemic disease stage. Using publicly available data, we found a significant enrichment of PU.1 binding sites in the preleukemic hypermethylated genes, suggesting that shortage of PU.1 makes PU.1 binding sites in the DNA accessible for aberrant methylation. Many known AML associated genes such as *RUNX1* and *HIC1* were found among the preleukemic hypermethylated genes. Nine novel hypermethylated genes, *FZD5*, *FZD8*, *PRDM16*, *ROBO3*, *CXCL14*, *BCOR*, *ITPKA*, *HES6* and *TAL1*, the latter four being potential PU.1 targets, were confirmed to be hypermethylated in human normal karyotype AML patients, underscoring the relevance of the mouse model for human AML.

**Conclusions:**

Our study identified early aberrantly methylated genes as potential contributors to onset and progression of AML.

## Background

Acute myeloid leukemia (AML) is an aggressive hematopoietic malignancy associated with severe morbidity and poor prognosis. It comprises a highly heterogeneous group of blastic myeloid malignancies and constitutes the most frequent type of acute leukemia in adults [1]. AML can arise *de novo* but also secondarily from preceding myelodysplastic syndrome (MDS), or after cytotoxic treatment or radiotherapy. It is characterized by an aggressive clonal proliferation of immature hematopoietic progenitor cells (myeloblasts) and impaired differentiation [2]. Recurrent chromosomal aberrations and rearrangements occur in more than 50% of cases and represent important predictive factors for response to therapy and outcome of the disease [3]. Altered gene function in AML is often a consequence of distinct cytogenetic aberrations [4], but also results from mutations in genes like *CEBPA* (*CCAAT/enhancer-binding protein*, *alpha*), *FLT3* (*fms-like tyrosine kinase receptor-3*), or *NPM1* (*nucleophosmin 1*) [3,4]. Although novel high-resolution genome-wide technologies have enabled the detection of numerous gene mutations, the multistep process of leukemogenesis is still poorly understood. In recent years, many reports have proposed that additional pathogenetic mechanisms, such as aberrant loss or gain of gene function due to epigenetic dysregulation, are of similar relevance for AML pathogenesis [5-8].

Methylation of cytosines in the context of CpG dinucleotides is a stable and common epigenetic modification in the mammalian genome. Most human gene promoters overlap with CpG-rich regions, designated CpG islands (CGIs), which are usually excluded from DNA methylation and, consequently, keep genes transcriptionally active. Conversely, promoter methylation is commonly linked with transcriptional silencing.

Hypermethylation and subsequent inactivation of genes are hallmarks of AML pathogenesis [9,10]. Prominent examples include the epigenetically silenced tumor suppressor genes *CDH1* or *p15/CDKN2B*[11,12]. In addition, gene hypomethylation is frequently found in myeloid malignancies. The mechanistic link, however, between promoter hypomethylation and tumorigenesis is incompletely understood. Global hypomethylation is common in many cancers, including AML, and is suspected to destabilize genome integrity by re-activating retrotransposons [6,13,14]. Alterations in DNA methylation contribute to initiation, expansion, and evolution of the leukemic clone and promoter hypermethylation is a frequent observation in specimens of patients with MDS and AML [15-17]. The mechanisms underlying the establishment of aberrant DNA methylation patterns are still largely unknown. Aberrant DNA methylation might be explained by the aberrant binding of transcription factors to their genomic target sequences. Transcription factor binding may prevent DNA methylation at these sequences, while diminished binding may result in *de novo* DNA methylation [18].

To gain a better insight into the molecular mechanisms and pathways underlying AML onset and progression, different mouse models recapitulating human AML have been generated, many based on the prevalent fusion genes *AML1*/*ETO*, *PML*/*RARA* or *MLL*/*ENL* (for review see [19]). These models mimic various human leukemogenic processes in the context of distinct disease genotypes and phenotypes associated with the different forms of AML [20].

Targeted deletion of an upstream regulatory element of the mouse gene *Sfpi1* (common human name *SPI1*) encoding the transcription factor PU.1, a key hematopoietic regulator for myeloid differentiation, results in homozygous PU.1 hypomorphs that develop AML (or much less frequently T-cell lymphomas) after a latency of 3 to 8 months [21,22]. As a consequence of reduced PU.1 expression in homozygous animals, epigenetic alterations of tumor suppressor genes are suspected to be involved in leukemogenesis. A previous screen of those animals developing a lymphoma but not an AML phenotype revealed promoter hypermethylation of the tumor-suppressor gene *Id4*[21].

Here, we provide a first comprehensive characterization of the methylome at CGIs in bone marrow (BM) cells from PU.1 hypomorphic animals during onset and progression of AML. Genome-wide DNA methylation screening during the process of leukemogenesis reveals the extent as well as the spatial and temporal distribution of altered DNA methylation. We aimed at identifying early differentially methylated genes preceding the fully established AML phenotype. Early differentially methylated genes may contribute to onset and progression of the disease, while the far more abundant and diverse differentially methylated genes at the late leukemic disease stage may reflect clonal diversification of AML and bystander events. We propose that hypomorphic PU.1 expression contributes to the initiation of aberrant DNA methylation of PU.1 target genes. Our study uncovers known and novel targets of aberrant epigenetic regulation occurring at onset and during progression of AML and, hence, may help to develop novel therapeutic strategies by revealing new pharmacologic targets at different stages of the disease.

## Methods

### Animals, sample collection and histopathology

Transcription factor PU.1 hypomorphic Balb/c mice with a homozygous deletion of an upstream regulatory element of gene *Sfpi1* encoding PU.1 were described previously [21,22]. Whole BM of age- and gender-matched homozygous wild-type (PU.1-wt) and PU.1 knockdown (PU.1-kd) mice were collected at three different disease stages. BM blast counts of PU.1-kd animals were used to define disease stages as preleukemic stage (BM blasts <20%, age 4 to 18 weeks, n = 7), early leukemic stage with residual non-malignant hematopoiesis (BM blasts between 20% and 50%, 4 to 12 weeks, n = 5) and late leukemic stage with full blown AML (>50%, 12 to 27 weeks, n = 7). From two of the seven animals of the late leukemic stage, the blast count could not be determined because the two mice died at the age of 22 to 24.5 weeks shortly before sampling. Both mice were considered late leukemic since they displayed phenotypic characteristics of their stage, such as enlarged spleens. BM cells were freshly collected from mouse femurs flushed with cold phosphate-buffered saline. Bone sections from forelegs were stained with hematoxylin and eosin and subjected to histopathological inspection for assessment of the disease stages.

Mouse BM cells were collected from the femorae, tibiae and iliae of PU.1-wt and preleukemic PU.1-kd mice by gentle crushing in Iscove’s modified Dulbecco’s medium. To confirm that the selected PU.1-kd animals were preleukemic, a May-Grünwald/Giemsa staining was performed on BM cytospins. The blast count was below 20% in each animal. Five PU.1-wt animals were pooled to obtain enough cells for sorting. For PU.1-kd animals, two groups with four preleukemic animals per group were collected. Murine lineage-depleted BM cells were isolated essentially as described in [23]. Low density mononuclear cells (LDMNCs) were purified by density gradient centrifugation using Histopaque 1083 (Sigma-Aldrich, Taufkirchen, Germany). LDMNCs were stained with the following rat anti-mouse biotin-conjugated lineage markers (all from BD Biosciences, Franklin Lake, NJ, USA): anti-CD5 (53-7.3), anti-CD8a (53-6.7), anti-CD11b (M1/70), anti-CD45R/B220 (RA3-6B2), anti-Ly-6G/Ly-6C (RB6-8C5) and anti-TER-119 (TER-119). The labeled LDMNCs were subsequently incubated with Biotin Binder Dynabeads (Life Technologies, Darmstadt, Germany) and the lineage-positive cells were depleted using a Dynamag-15 magnet, resulting in lineage-depleted cells. The lineage-depleted cells were stained with the following panel of antibodies: FITC-conjugated rat anti-mouse CD34 (RAM34; eBioscience, Frankfurt, Germany); eFlour®450-conjugated rat anti-mouse CD16/32 (93, eBioscience); APC-conjugated rat anti-mouse CD127 (A7R34, eBioscience); PE-conjugated rat anti-mouse CD117/c-Kit (2B8, eBioscience); APC-Cy7-conjugated rat anti-mouse Ly-6A/E/Sca-1 (D7; BD Biosciences, Heidelberg, Germany); and PE-Cy7-conjugated Streptavidin (eBioscience). Lineage-negative, CD127-, c-Kit+, Sca-1- cell fractions corresponding to granulocyte-macrophage progenitor cells (GMPs; CD16/32+, CD34+), common myeloid progenitor cells (CMPs; CD16/32-, CD34+), and megakaryocyte-erythroid progenitor cells (MEPs; CD16/32-, CD34-) as well as the lineage-negative, CD127-, c-Kit+, Sca-1+ cell (LSK) fraction were then prospectively isolated using a BD FACSAria I, II or III flow cytometer (BD Biosciences). All animal experiments were performed in accordance with the institutional guidelines of the German Cancer Research Center and were approved by the Regierungspräsidium Karlsruhe, Germany.

MDS patient samples from whole BM (n = 149) and from peripheral blood (n = 1) reflecting the entire disease spectrum as displayed by the World Health Organization (WHO) classification were obtained from the Department of Hematology, Oncology and Clinical Immunology, Heinrich-Heine University, Düsseldorf, Germany with patient informed consent and the University Clinic Düsseldorf review board approval in accordance with the Declaration of Helsinki. The sample set contained the following MDS subgroups: MDS with del(5q) (5q-, n = 5), refractory anemia (RA, n = 6), refractory anemia with ringed sideroblasts (RARS, n = 8), refractory cytopenia with multilineage dysplasia (RCMD, n = 43), refractory cytopenia with multilineage dysplasia and ringed sideroblasts (RCMD-RS, n = 30), refractory anemia with excess of blasts type I and II (RAEBI, n = 18; RAEBII, n = 28), chronic myelo-monocytic leukemia type I and II (CMMLI, n = 10; CMMLII, n = 2). Normal karyotype AML patient samples from whole BM (n = 46) and from peripheral blood (n = 5) were obtained from the Department of Hematology/Oncology, University Medical Center, Freiburg, Germany with patient informed consent and the University Clinic Freiburg review board approval in accordance with the Declaration of Helsinki. Healthy granulocytes were isolated from blood of 14 healthy donors using Leukosep (Greiner bio-one, Frickenhausen, Germany) according to the manufacturer's instructions. CD34+ cells were from two healthy female and one healthy male donor (median age 36 years) and purchased from Lonza (Verviers, Belgium).

### DNA and RNA extraction

DNA and RNA were extracted from mouse BM using the Allprep Mini Kit (QIAGEN, Hilden, Germany) according to the manufacturer’s protocol and stored at 4°C (DNA) or -80°C (RNA).

DNA of human MDS and AML samples was isolated with the QIAmp DNA Mini Kit (QIAGEN) according to the manufacturer’s instructions.

### Methyl-CpG immunoprecipitation

Methyl-CpG immunoprecipitation (MCIp) was performed as described previously [24]. In brief, a total of 2.5 μg DNA was sonicated with the Bioruptor NextGen (Diagenode, Liege, Belgium) to fragments of 100 to 600 bp as monitored on a 1.5% agarose gel. MCIp enrichment of highly methylated DNA was performed, as described, with minor modifications using SX-8G IP-Star robot (Diagenode). Sonicated DNA was enriched with 90 μg purified methyl-CpG-binding domain-Fc protein coupled to 60 μl protein A-coated magnetic beads (Diagenode). DNA was eluted by incubation with increasing NaCl concentrations (fraction A, 300 mM; B, 400 mM; C, 500 mM; D, 550 mM; E, 1,000 mM). Desalted eluates were controlled for enrichment of methylated DNA by real-time PCR analyzing the imprinted gene *Mest*. The non-methylated allele elutes at low-salt while the methylated allele elutes at high-salt concentration.

### Methylome profiling by microarray analysis

Highly methylated DNA, corresponding to fraction E of the enrichment procedure, from age- and gender-matched PU.1-kd and PU.1-wt animals was labeled with Alexa 3 (PU.1-wt) or Alexa 5 (PU.1-kd) and co-hybridized to a mouse CGI array (Agilent, Böblingen, Germany) covering the about 16,000 CGIs, represented by 88,358 probe sequences with a length of 45 to 60 bp per probe sequence, of the mouse genome (approximately 0.4%; NCBI36/mm8). Henceforth, probe sequences are designated 'probes' throughout this study and 'differentially methylated probes' (DMPs), if they were differentially methylated between PU.1-kd and PU.1-wt animals. Agilent’s annotation, according to NCBI36/mm8, assigned the probes to 5,285 gene promoters, 7,872 gene bodies, 480 locations downstream of genes and 1,869 locations with unknown genomic feature. Here, promoter probes were defined as those being located 2,000 bp upstream to 500 bp downstream of a gene's transcription start site. In all other cases, we followed the annotation of Agilent. Microarrays were analyzed using a DNA microarray scanner (Agilent) and Feature Extraction Software 10.5 (Agilent) with the ChIP protocol setting. Data processing and statistical analyses were done within the *R* statistical environment, v. 2.13.1 [25]. Background correction and log 2-ratio transformation were performed according to the NormExp method with offset = 50; any intensity that is less than 0.5 after background subtraction is reset to be equal to 0.5 [26]. Variation between co-hybridized samples was reduced by intensity-based LOESS normalization on rank-invariant probes and negative controls [27].

The CGI array data of this study has been deposited at the NCBI Gene Expression Omnibus [28] under accession number GSE37315.

### Quantitative DNA methylation analysis

The degree of DNA methylation was determined by MALDI-TOF mass spectrometry (MassARRAY, Sequenom, San Diego, USA) as previously described [29]. Amplicon primers (Additional file 1) flanked genomic stretches that covered at least one of the hypermethylated probes represented on the CGI array. *In vitro* methylated standard DNA served as control. Unmethylated DNA was produced using the REPLI-g Mini Kit (QIAGEN) and purified with the QIAmp DNA Mini Kit (QIAGEN). Half of the unmethylated DNA was methylated using M.SSSI enzyme and purified with the QIAquick gel extraction kit (QIAGEN). Unmethylated and methylated DNA was mixed to obtain different ratios for the standard DNA (0%, 20%, 40%, 60%, 80% and 100% DNA methylation).

### Overlap of publicly available chromatin immunoprecipitation sequencing data with methylome data

Genomic coordinates from publicly available chromatin immunoprecipitation sequencing (ChIP-Seq) data of transcription factor PU.1 (NCBI37/mm9) [30] were converted to the NCBI36/mm8 (2006) genome by using the lift over tool of the UCSC Genome Browser [31]. In total, 22,625 out of 22,720 peak regions could be successfully converted. The PU.1 ChIP coordinates were overlapped with genomic coordinates of the preleukemic hypermethylated genes, and Fisher's exact test was used to test for significant enrichment of the PU.1 ChIP peaks. We randomly permutated the PU.1 ChIP-Seq peaks 1,000 times over the genomic coordinates of the preleukemic hypermethylated CGIs and compared the randomly permutated numbers with the actual numbers of PU.1 ChIP-Seq peak/preleukemic hypermethylated CGI overlap.

### Statistical analysis

Following CGI array normalization, one class significance analysis of microarrays (SAM; package samr, version 2.0 [32]), was performed for each disease stage separately to find significantly hyper- and hypomethylated probes between PU.1-kd and PU.1-wt mice with a false discovery rate of 5%. Principal component analysis (PCA; package pcaMethods, version 1.36.0) was performed using the array methylation patterns to analyze homogeneity between individual animals at the molecular level in the course of the disease.

Unsupervised clustering with Euclidian distance and the average agglomeration method was used for clustering MassARRAY data. Unsupervised clustering was performed by multiscale bootstrap resampling [33] (package pvclust, version 1.2.2) to calculate approximately unbiased (AU) *P*-values for each cluster in the result of hierarchical clustering. The number of permutations was set to 1,000.

Mann-Whitney U test was performed to test for methylation differences derived from MassARRAY data between PU.1-kd and PU.1-wt animals and between AML/MDS samples and healthy granulocytes/CD34+ cells.

The computational analysis was performed by custom Perl scripts and the motif discovery was conducted with the software suite HOMER (Hypergeometric Optimization of Motif EnRichment) [34].

### Pathway analysis

Pathway analysis was performed using genes that were represented by at least two DMPs of the preleukemic stage through the use of Qiagen’s Ingenuity® Pathway Analysis [35].

## Results

### Disease progression is associated with alterations in global DNA methylation

**Figure 1:**
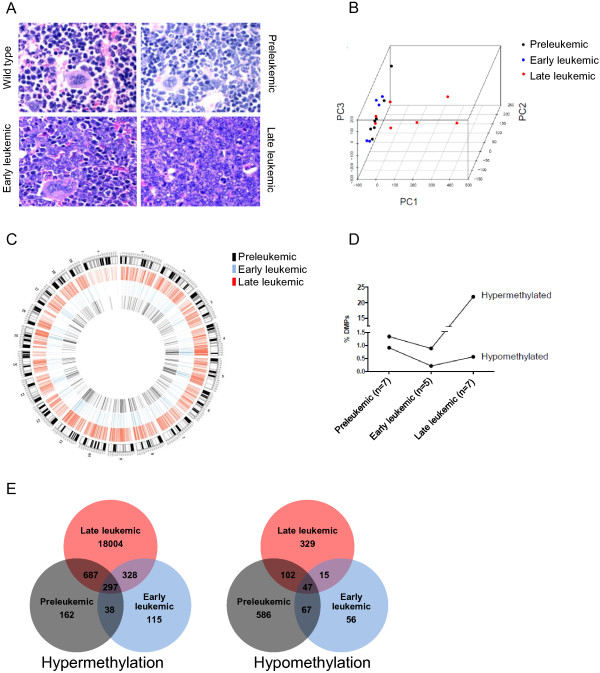
**Disease progression and accompanying global DNA methylation in the PU.1 mouse model. (A)** Representative histological sections of mouse forelegs showing BM of PU.1-wt and PU.1-kd animals of different disease stages (preleukemic stage, BM blasts <20%; early leukemic stage with residual non-malignant hematopoiesis, BM blasts between 20% and 50%; late leukemic stage with full blown AML, BM blasts >50%). Sections were stained with hematoxylin and eosin; 400× original magnification. **(B)** PCA based on normalized relative probe intensities between PU.1-kd versus PU.1-wt animals. In total, 88,358 array probes per sample were analyzed. Principal component (PC)1 explains the largest variances of the entire data set and implicates diversification of the DNA methylation patterns in late leukemic stage animals. **(C)** Circos plot showing the hypermethylated probes of the three disease stages (preleukemic, early leukemic, late leukemic). The outer circle indicates the G-banded mouse chromosomes, the differently colored lines in the inner circles represent hypermethylated probes of the three stages. **(D)** Percentage of hyper- and hypomethylated probes (DMPs) in the different disease stages (preleukemic, early leukemic, late leukemic). SAM was performed to identify the DMPs. Out of 88,358 probes, 20,787 were aberrantly methylated in at least one disease stage. **(E)** Venn diagrams showing unique and common hyper- and hypomethylated probes in different disease stages.

In order to determine DNA methylation changes in the progression of leukemic cells, we used the murine AML model driven by hypomorphic expression of the hematopoietic transcription factor PU.1 and MCIp as a screening tool. MCIp allows the enrichment of methylated DNA from cell populations and, in this way, is conceptually different to whole genome bisulfite sequencing. We monitored the proportion of BM blasts in PU.1 hypomorphic mice (PU.1-kd) that develop an AML-like malignancy. According to blast counts, we defined the three disease stages as preleukemic (BM blasts <20%, n = 7, median age 4 to 18 weeks), early leukemic (BM blasts 20 to 50%, n = 5, 4 to 12 weeks) and late leukemic stage (BM blasts >50%, n = 7, 12 to 27 weeks) (Figure 1A). While preleukemic and early leukemic stage animals were phenotypically inconspicuous, late leukemic stage animals displayed pronounced morbidity and suffered from eye and ear infections and from massive spleno- and hepatomegaly as previously described [22]. Using CGI tiling microarrays, we cohybridized MCIp-enriched, highly methylated DNA of the PU.1-kd animals with that of age- and gender-matched PU.1-wt. Similarity between the PU.1-kd animals with respect to the DNA methylation patterns was tested by PCA using the signal intensity ratio (M-value) of the array probes as a measure for enrichment (Figure 1B). The largest variance as explained by principal component 1 (PC1) was observed for individuals of the late leukemic disease stage that clearly separated them from preleukemic and early leukemic stage animals. In addition, late leukemic stage animals showed a more diverse distribution, suggesting a larger inter-individual heterogeneity of DNA methylation patterns; preleukemic and early leukemic stage animals grouped together.

### Early aberrant DNA methylation is followed by the diversification of hypermethylation in the late leukemic disease stage

We used the array-derived M-values to characterize the three disease stages by SAM. Of the 88,358 probes, 20,787 (23.5%) were aberrantly methylated in at least one stage of the disease. Aberrant DNA methylation was equally distributed across the genome, and no chromosome was preferentially hypermethylated (Figure 1C) or hypomethylated (Additional file 2). In the preleukemic stage, 1,184 (1.34%) hyper- and 802 (0.91%) hypomethylated probes were found, covering 762 and 504 genes or other genomic locations, respectively (Additional file 3). Both numbers slightly decreased in the early leukemic stage, suggesting either partial reversion of initial methylation changes or concomitant molecular processes other than aberrant DNA methylation (for example, genetic alterations) driving malignant clone selection (Figure 1D). The number of hypermethylated, but not that of hypomethylated probes abruptly increased in the late leukemic stage to 19,316 (21.9%), covering 5,465 genes or other genomic locations (Figure 1D). Within the respective stages, several unique hyper- and hypomethylated probes were observed. For example, 162 probes were uniquely hypermethylated in the preleukemic stage, but were unchanged or even hypomethylated in the other stages. Throughout all stages, 297 (0.3%) probes were commonly hypermethylated and 47 probes were commonly hypomethylated (Figure 1E).

**Figure 2:**
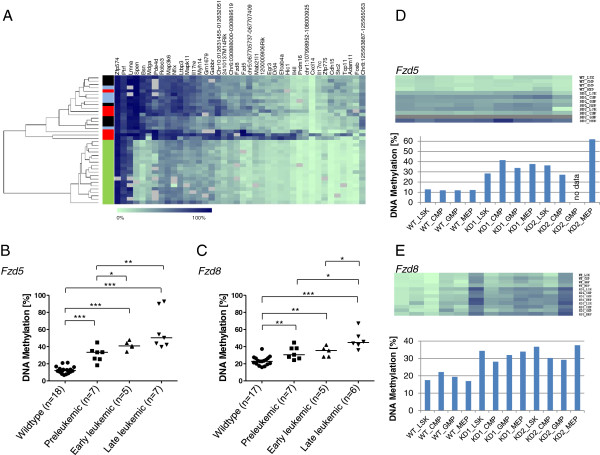
**Validation of screening results by quantitative DNA methylation analysis (MassARRAY). (A)** Heatmap showing the average methylation levels in 40 gene-specific amplicons (columns) and individual PU.1-kd and PU.1-wt animals (rows). Methylation levels range from 0% (light green) to 100% (dark blue). Grey boxes represent missing values. The bar to the left of the heatmap indicates different disease stages (black, preleukemic; blue, early leukemic; red, late leukemic; green, PU.1-wt). Unsupervised clustering discriminates PU.1-kd and PU.1-wt animals. **(B,C)** DNA methylation levels of *Fzd5 ***(B)** and *Fzd8 ***(C)**. Average amplicon methylation is shown for different animals of the different stages. The black bar represents the median methylation within one stage. Mann-Whitney U test was used to test for differences between the different disease stages (*P < 0.05, **P < 0.01, ***P < 0.001). **(D,E)** Methylation levels (heatmaps on top, bar graphs below) of *Fzd5 ***(D)** and *Fzd8 ***(E)** amplicons in sorted cells of PU.1-wt animals and two groups of preleukemic PU.1-kd animals, KD1 and KD2. The sorted cells comprise LSKs (lineage-negative, Ska1-positive, c-kit-negative cells), CMPs (common myeloid progenitor cells), GMPs (granulocyte-macrophage progenitor cells) and MEPs (megakaryocyte-erythroid progenitor cells). The heatmaps display single CpG units (columns) of PU.1-kd or PU.1-wt animals (rows). Methylation values range from 0% (light green) to 100% (dark blue). The bar graphs show average methylation (y-axis) of the different amplicons. In **(D)**, analysis of KD2-GMP failed, indicated by grey CpG units in the heatmap and missing value in the bar graph.

We randomly selected 40 hypermethylated genes of the preleukemic stage for technical validation by quantitative high-resolution methylation analysis using MassARRAY. For 34 genes (85%), hypermethylation in preleukemic versus matched PU.1-wt animals was confirmed (*P* < 0.05; Figure 2A; Additional file 4) and, hence, indicated high reliability of our CGI array data. Hypermethylation was also confirmed for 36 (90%) in the early leukemic and for 37 (92.5%) in the late leukemic stage. Moreover, unsupervised clustering using the quantitative methylation data recapitulated the results of the global PCA-based analysis and discriminated the PU.1-kd from the PU.1-wt animals (Figure 2A).

We observed a significant gradual increase of methylation levels with disease progression in the majority of analyzed genes as exemplified by *Fzd5* and *Fzd8* (Figure 2B,C). *Fzd5* methylation was 12.1% (range 6.8 to 21.3%) in PU.1-wt animals and 33.4% (range 18.2 to 44.9%), 40.9% (range 34 to 47.8%) and 50.4% (range 39.8 to 92.8%) in preleukemic, early leukemic and late leukemic stage animals, respectively. The stage-dependent increase of *Fzd8* methylation was similar to that of *Fzd5*, starting with 22.8% (range 15.9 to 37.3%) in PU.1-wt animals and increasing to 30.6% (range 24.3 to 44.7%), 35.4% (range 28.1 to 41.9%) and 44.8% (range 36.1 to 67.2%) in the preleukemic, early leukemic and late leukemic stage animals, respectively.

### Acute myeloid leukemia-specific methylation changes

To ensure that the observed changes in DNA methylation are not the result of tissue-specific methylation patterns and, thus, reflect differences in cell type composition, we analyzed *Fzd5*, *Fzd8* and eight additional differentially methylated regions in four hematopoietic cell types, LSK (lineage-negative, c-Kit+, Sca-1+ cells), CMPs, GMPs and MEPs, which were enriched from both PU.1-wt and PU.1-kd BM, respectively, and which represent different stages of hematopoietic commitment. We found both genes to be similarly hypermethylated in all four cell types in PU.1-kd BM (Figure 2D,E; Additional file 5). This indicates that hypermethylation at these loci is an early event that is specific for PU.1-kd and does not just reflect the expansion of specific hematopoietic compartments. In summary, our quantitative methylation data indicate dynamic changes from onset to the late leukemic stage of the disease. The correlation between DNA methylation level and myeloblast infiltration suggests that aberrant DNA methylation is a feature of the malignant clone. Early aberrant DNA methylation at specific loci, in turn, can ubiquitously be found in the myeloid compartment and may characterize the (pre)-malignant clone in its early stage.

### Early targets of aberrant DNA methylation in the PU.1 mouse model are relevant for the pathogenesis of human myeloid malignancies

To identify genes potentially involved in the onset of AML, we looked for overlaps between the list of 1,229 genes or other genomic locations indicated by aberrantly methylated probes in the preleukemic stage (Additional file 3) and gene lists from previously published genome-wide DNA methylation data derived from the HELP (*Hpa*II tiny fragment enrichment by ligation-mediated PCR) assay in human MDS and AML [17]. We detected 291 common genes with MDS-associated DNA methylation targets (5,390 in total) and 30 common genes with the AML gene list (475 *de novo* targets in total) (Additional file 6). These overlaps corroborate the relevance of the animal model for acute human myeloid malignancies. Well-known examples of human leukemogenesis, such as *RUNX1*, *CEBPA*, and *ABL1*, were aberrantly methylated in both the murine preleukemic stage and in human MDS. Remarkably, the ratios between overlapping genes and the aberrantly methylated human disease genes (291/5,390 ~ 0.05 for MDS and 30/475 ~ 0.06 for AML) were rather similar, suggesting that the mouse model was not confined to only a distinct disease type of either MDS or AML.

We tested by gene ontology analysis (Ingenuity Pathways Analysis) whether the set of aberrantly methylated, preleukemic genes represented functional groups of genes or pathways relevant for AML pathogenesis. Among several partially overlapping signaling pathways, Wnt/β-catenin and embryonic stem cell signaling were prominently overrepresented (Additional file 7). These pathways are known to be involved in onset and progression of human malignancies, including AML [36,37]. Moreover, Wnt signaling has already been linked to the inappropriate regulation of the PU.1 transcription factor associated with T-cell lymphoma in mice [21].

**Figure 3:**
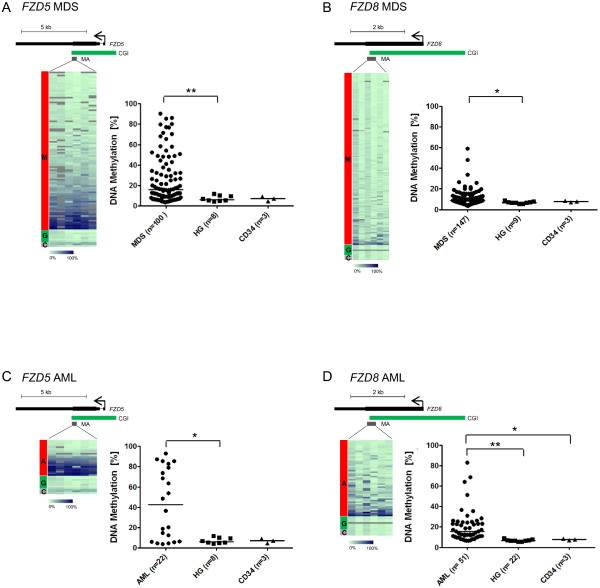
**Hypermethylation of *FZD5 *and *FZD8 *in MDS and AML patients. (A-D)** Heatmaps and dotplots of amplicons from *FZD5 ***(A,C)** and *FZD8 ***(B,D)** in MDS (top) and AML (bottom) patients compared to healthy granulocytes/CD34+ cells. The heatmaps display methylation levels of single CpG units (columns). Methylation values range from 0% (light green) to 100% (dark blue). Differently colored bars to the left of the heatmaps indicate MDS (M), AML (A), healthy granulocytes (G) and CD34+ cells (C). Schemes above the heatmaps display the gene (black bar), transcription start (arrow), the relative location of the CpG island (CGI) and the amplicon analyzed (MA). The dotplots show average amplicon methylation levels of individual MDS/AML patients, healthy granulocytes (HG) and CD34+ cells (CD34). The median methylation in a group is depicted by a black bar (median methylation of *FZD5* was 15.7% in MDS samples and 42.5% in AML samples; median methylation of *FZD8* was 8.8% in MDS samples and 15.5% in AML samples). Mann-Whitney U test was used to test for differences between MDS/AML samples, healthy granulocytes and CD34+ cells (*P < 0.05, **P <0.01).

Since the Wnt signaling genes *Fzd5* and *Fzd8* showed enhanced CGI hypermethylation with increasing disease stage (Figure 2B, C), we examined the methylation state of the homologous human CGI sequences in sets of MDS (15.7% and 8.8% median DNA methylation for *FZD5* and *FZD8*, respectively) and normal karyotype AML (42.5% and 15.5%) patient samples. Despite large methylation ranges in the patient samples, both genes proved significantly hypermethylated in both MDS and AML compared to granulocytes (6.3% and 6.7%) and CD34+ cells (25.6% and 7.7%) from healthy donors; hypermethylation was more pronounced in the latter (Figure 3). No significant difference could be detected between different risk groups in the MDS patients according to IPSS (International Prognostic Scoring System, consisting of the fraction of BM blasts, number of cytopenias and the cytogenetic risk group) or to WHO classification subgroups combined by blast count range (Additional file 8).

We selected three additional genes, *PRDM16*, *ROBO3*, and *CXCL14*, that displayed promoter hypermethylation already in the preleukemic or early leukemic stage (Additional file 4) for validation in human AML samples. Thus far, these genes have not been shown to be aberrantly methylated in human AML; however, *PRDM16* and *ROBO3* are differentially methylated in MDS [17]. *PRDM16* is a fusion partner of *RPN1*, *RUNX1*, and other genes in hematopoietic malignancies [38,39], and rearrangement of *PRDM16* was associated with poor prognosis [38]. *ROBO3* is hypermethylated in cervical cancer [40]. *CXCL14* is important in the progression of many malignancies, including colorectal cancer [41], and is epigenetically silenced in lung and prostate cancer [42,43]. All three genes showed promoter hypermethylation in the AML samples, and that of *ROBO3* and *CXCL14* reached statistical significance (*P* < 0.05; Additional file 9).

### Loss of PU.1 binding contributes to aberrant DNA methylation

**Figure 4:**
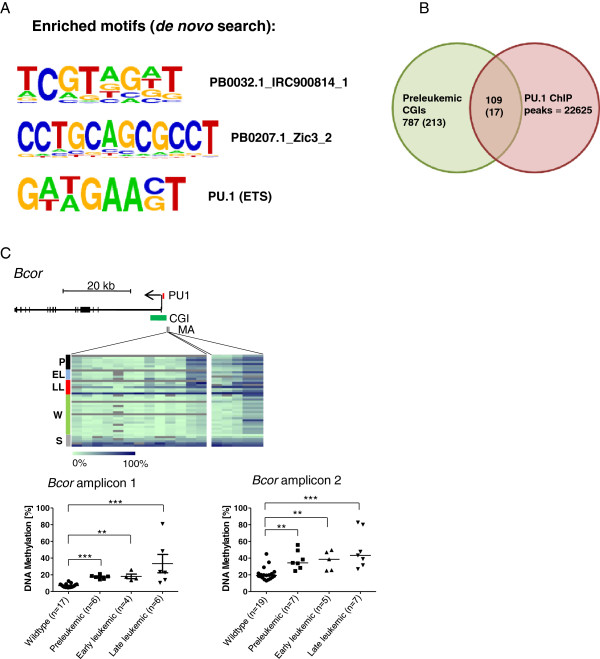
**PU.1 motifs and binding peaks are enriched among the preleukemic hypermethylated genes. (A)** Enriched sequence motifs within the preleukemic hypermethylated genes. **(B)** Venn diagram showing the number of overlapping and non-overlapping genomic coordinates of preleukemic hypermethylated CGIs (green) and publicly available ChIP-Seq data (red). Numbers in brackets represent a more stringent analysis, namely preleukemic hypermethylated genes represented by at least two hypermethylated probes. **(C)** Validation of PU.1 target gene *Bcor* by MassARRAY. Heatmaps display methylation levels of single CpG units (columns) in different disease stages (rows). Differently colored bars to the left of the heatmaps display preleukemic (P, black), early leukemic (EL, blue), late leukemic (LL, red), wild type (W, green) and standard (S, grey). Methylation values range from 0% (light green) to 100% (dark blue). The scheme above the heatmaps displays the gene (black bar), transcription start (arrow), the PU.1 ChIP peak (PU1), the relative location of the CpG island (CGI) and the two MassARRAY amplicons (MA). The dotplots below the heatmaps show the average amplicon methylation levels of wild-type animals and different disease stages. The median methylation in a sample group is indicated by a black bar. Mann-Whitney U test was used to test for differences between wild type and stages and between stages (*P < 0.05, **P <0.01, ***P < 0.001).

Transcription factors bound to their genomic target sequences may prevent DNA methylation at these sequences, whereas reduction or loss of transcription factor binding may result in *de novo* DNA methylation [18]. We hypothesized that hypomorphic expression of the PU.1 transcription factor entails reduced DNA binding of PU.1; this reduction, in turn, may contribute to aberrant DNA methylation patterns of PU.1 target genes. Therefore, we searched for overrepresented sequence motifs within all preleukemic hypermethylated CGIs (787) and found a significant overrepresentation of a PU.1 binding motif among these CGIs (*P* = 1e-11; Figure 4A). Furthermore, the search for known binding factors revealed a significant overrepresentation of binding sites for the E2f family and for FoxA1 (*P* = 0.01; Additional file 10), the former known to contribute to hematopoiesis [44], the latter known to be involved in normal and cancer development [45]. An additional search for the consensus PU.1 binding motif GAGGAA in the complete mouse genome (mm8, 2006) revealed 704,291 sites of which 99 overlapped with the preleukemic hypermethylated CGIs. Random permutation of the PU.1 motif resulted in an average of only 57 matches, indicating that the PU.1 motif is enriched in the preleukemic hypermethylated CGIs (Fisher's exact test, *P* < 0.001). Taken together, our motif search revealed a variety of binding sites for known transcription factors relevant in hematopoiesis, but also novel motifs of yet unknown function.

To identify PU.1 target genes associated with the preleukemic hypermethylated CGIs, we searched for overlaps between the genomic coordinates of PU.1 binding sites obtained from publicly available PU.1 ChIP-Seq data [30] and the coordinates of the preleukemic hypermethylated CGIs. We found an overlap of 109 genes or other genomic locations (Figure 4B). In a more stringent search, we selected only the preleukemic CGIs that were covered by at least two hypermethylated probes. Here, 17 from 214 preleukemic hypermethylated CGIs overlapped with a PU.1 ChIP peak (Figure 4B; Additional file 11). Enrichment of PU.1 targets among the preleukemic hypermethylated genes was significant for both search stringencies (*P* < -2.2e-16 for 109/787 genes and *P* = 0.002049 for 17/214 genes). From the 17 genes identified under higher stringency, we selected three gene promoters, *Bcor*, *Itpka*, and *Hes6*, for validation by quantitative methylation analysis in PU.1-kd and -wt animals. *Bcor* mutations have been found in AML [46], *Itpka* contributes to differentiation of human embryonic stem cells [47] and is downregulated in oral squamous cell carcinoma [48], and *Hes6* is overexpressed in glioma and breast cancer [49,50]. From those overlapped regions only covered by a single hypermethylated probe (109; Figure 4B), we selected a fourth gene, *Tal1*, a known PU.1 target, because of its function in normal hematopoiesis and leukemogenesis [51,52]. Hypermethylation of all four PU.1 target genes was confirmed by MassARRAY in the PU.1-kd samples (Figure 4C; Additional file 12). We also analyzed the genes in four hematopoietic cell types, LSK, CMP, GMP and MEP. We found all genes similarly methylated in all cell types (Additional file 5).

**Figure 5:**
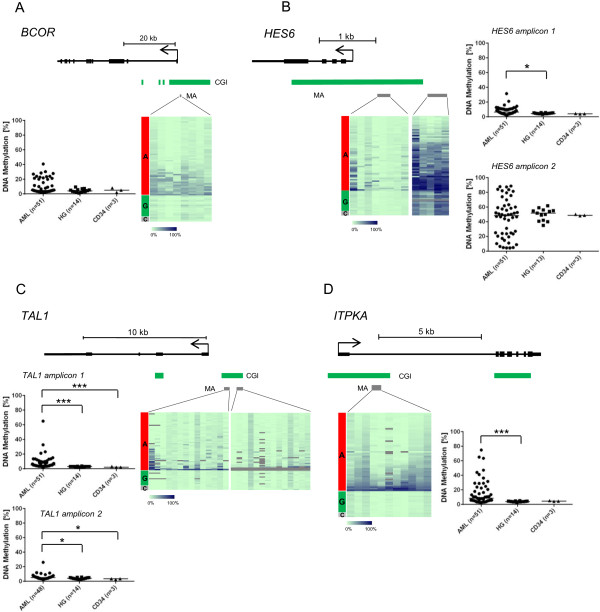
**Hypermethylation of PU.1 target genes in AML samples. (A-D)** Validation of four PU.1 target genes, *BCOR ***(A)**, *HES6 ***(B)**, *TAL1 ***(C)** and *ITPKA ***(D)**, by MassARRAY in human AML samples compared to healthy granulocytes (HG) and CD34+ cells. For details, refer to the legend of Figure 3. Median methylation values in AML samples are as followed: *BCOR*, 4.6%; *HES6* amplicon 1, 5.6%; *HES6* amplicon 2, 49.7%; *TAL1* amplicon 1, 4.2%; *TAL1* amplicon 2, 4.6%; ITPKA, 5.9% (*P < 0.05, ***P <0.001).

Of the four PU.1 target genes, *BCOR*, *ITPKA* and *TAL1* were also found aberrantly methylated in a recent genome-wide screen of MDS patients [17]. We examined the methylation levels of the four genes in human AML samples and observed significant hypermethylation compared to healthy granulocytes and CD34+ cells in *HES6*, *ITPKA* and *TAL1*, while *BCOR* showed a trend towards hypermethylation in a subgroup of AML patients (Figure 5). Additionally, we could relate PU.1 mRNA expression with target gene methylation in a set of 26 AML patients, where the expression differed by a factor of up to six-fold. However, no correlation between PU.1 mRNA expression and methylation at presumed PU.1 binding sites in the promoters of the four genes showed up (data not shown).

Taken together, our results suggest that loss of PU.1 binding contributes to the establishment of aberrant DNA methylation patterns. Similarities between DNA methylation patterns of the PU.1-kd mice and human MDS and AML patients underscore the benefit of studying early epigenetic changes in the mouse model for the identification of genes potentially involved in onset and progression of the disease. We present various novel genes associated with AML as potential early targets for aberrant DNA methylation during leukemogenesis.

## Discussion

AML pathogenesis is a complex multistep process that involves an interplay of genetic and epigenetic aberrations. The time from disease onset to its full-blown clinical picture requires detailed knowledge about the timing of disease-driving molecular mechanisms to successfully interfere with these processes by targeted therapy. Here, we addressed the question whether epigenetic aberrations already contribute to the early events and processes in AML pathogenesis by utilizing a murine AML progression model with a stable down-regulation of the hematopoietic transcription factor PU.1 [22]. We characterized DNA methylation dynamics over three stages of disease development and demonstrated that distinct DNA methylation changes occur early and subsequently expand during leukemogenesis. The reliability and relevance of methylation assessment by our genome-wide, array-based approach was corroborated by independently confirming 34 of 40 selected genes/chromosomal locations using quantitative, high-resolution mass spectrometry.

PCA with the DNA methylation values of all CGI array probes distinguished late leukemic stage from preleukemic and early leukemic stage animals. Preleukemic animals with low or absent myeloblasts already exhibited a high number of hypermethylated sequences, indicating extensive involvement of epigenetic mechanisms at this stage. These sequences represented 762 genes or other genomic locations. Approximately one-fourth of the preleukemic hypermethylated sequences were consistently hypermethylated throughout all leukemogenic stages, underscoring the pathogenic relevance of the affected genes for disease initiation and progression. Compared to hypermethylation, hypomethylation was considerably less abundant, probably due to its preferential occurrence outside of CGIs. Thus, our findings highlight that CGI hypermethylation accompanies AML onset and, therefore, may contribute to AML development.

From the preleukemic to the early leukemic stage, the number of hypermethylated probes appeared largely stable. At the late leukemic stage, however, the number of hypermethylated probes strikingly increased approximately 20-fold, accompanied by genomic diversification of DNA methylation. Cluster analysis of quantitative methylation values clearly discriminated between PU.1-wt and PU.1-kd animals.

The increase of aberrant DNA methylation abundance in the late leukemic stage cannot simply be explained by the mere increase in blast counts, but might rather be the consequence of a vigorous 'epigenetic' clonal evolution or of severe disturbance of the epigenetic machinery. When we examined the methylation levels in diverse hematopoietic progenitors at the preleukemic stage, all cell types displayed hypermethylation, similar to that of the bulk of myelogenic cells at this stage. Accordingly, we could so far neither dissect the AML cell of origin nor attribute leukemic expansion to the expansion of a distinct hematopoietic lineage.

Our study supports a model of an epigenetic outburst targeting distinct regions early in disease progression. This could be a consequence of genetic alterations in enzymes regulating epigenetic patterns, such as gene mutations found in human myeloid malignancies, including *DNMT3a*, *TET2*, *IDH1*, *IDH2*, *EZH2*, or *ASXL1*[53]. With respect to the dramatic outburst of aberrant DNA methylation in the late stage, our AML-like mouse model differs from a recently reported chronic lymphocytic leukemia-like mouse model, where early DNA methylation events are followed by a gradual increase of aberrantly hypermethylated genomic regions over time [54].

We identified a wealth of known and novel AML-associated genes, epigenetically altered already at the preleukemic disease stage, and provide a repository of 762 early hypermethylated and 504 hypomethylated genes, together constituting a valuable resource for investigating potential key pathogenic factors in AML. Since methylation of cytosine is a reversible epigenetic modification, and demethylating drugs are already used in the clinical setting for treatment of both MDS and AML patients [55,56], the novel early candidates identified in this study may point towards druggable mechanisms and pathways for targeted therapy. In line with observations by others [36,37], a prominent role at disease onset can be ascribed to the Wnt signaling pathway, since members of this pathway, *Fzd5*, *Fzd8*, *Fzd10*, and *Wnt3* (Additional file 7), were overrepresented among the early aberrantly methylated targets. The link between Wnt signaling and the PU.1-kd-driven AML mouse model is corroborated by earlier observations that PU.1 is targeted by Wnt pathway members [21].

We detected a considerable overlap between early aberrantly methylated genes and genes involved in human myeloid malignancies (MDS and AML) [17], indicating the relevance of the observed epigenetic changes in the mouse model for human disease. Hypermethylated genes in the preleukemic stage such as *Cebpa* and *Hic1* have already been described as being hypermethylated as well in AML [57,58]. Moreover, normal karyotype AML and MDS patients (of different WHO subtypes) displayed hypermethylation of the Wnt pathway members *FZD5* and *FZD8*, as observed in the mouse model. We confirmed three additional candidates, *PRDM16*, *ROBO3* and *CXCL14*, to be hypermethylated in the AML patient cohort. So far, none of these five genes has been validated as being aberrantly methylated in AML by a quantitative high resolution method, albeit *FZD5*, *FZD8*, *ROBO3* and *PRDM16* have been found in other genome-wide methylation screens of MDS samples [17]. The concordant presence of aberrant methylation in these candidate genes already in early stages of our mouse model as well as in both MDS and AML suggests a disease driving potential of these aberrations.

It has been shown previously that binding of transcription factors to target DNA sequences may prevent their methylation [18]. In line with this, knockdown of transcription factor PU.1 was associated with preleukemic hypermethylation at a considerable number of PU.1 target sequences derived from publicly available ChIP-Seq data [30]. Looking closer at four selected PU.1 target genes by quantitative methylation analysis, we confirmed hypermethylation in both PU.1-kd animals and human AML samples. However, a correlation between PU.1 mRNA expression and methylation levels of the selected target genes *BCOR*, *HES6*, *ITPKA* and *TAL1* could not be demonstrated in AML patients, suggesting other mechanisms than mere PU.1 down-regulation to be required for hypermethylation of these genes in human AML.

Taken together, our results suggest that the PU.1-kd mouse is a valuable model to study epigenetic changes during AML progression. The newly identified early hypermethylated genes are potential determinants for aberrant DNA methylation patterns in the disease course and, consequently, may contribute to disease development in humans. Early epigenetic changes are suspected drivers of malignancies and, hence, may offer the chance to identify suitable drug targets for early therapeutic intervention. As shown here, epigenetic profiling of tumor progression models is a promising strategy to highlight the role of epigenetics in disease initiation and progression.

## Conclusions

In the present study, we utilized a mouse model of leukemogenesis to identify epigenetically altered genomic loci on a global scale and to determine the timing of altered epigenetic reprogramming. DNA methylation profiling of the PU.1 mouse model of leukemogenesis enabled detailed insight into the extent and dynamics of aberrant epigenetic mechanisms and created a valuable resource of early aberrantly methylated genes. We demonstrate that DNA methylation changes occur along AML pathogenesis in mice, and that these specific alterations recapitulate the alterations seen in human myeloid malignancies. Thus, this mouse model represents a suitable tool to investigate the molecular mechanisms leading to epigenetic reprogramming. We found high numbers of genes affected by epigenetic changes. The wealth of early affected loci strongly argues for a prominent role of epigenetic mechanisms in the pathogenesis and progression of MDS and AML.

## Abbreviations

AML: acute myeloid leukemia; BM: bone marrow; bp: base pair; CGI: CpG island; ChIP: chromatin immunoprecipitation; CMML: chronic myelo-monocytic leukemia; CMP: common myeloid progenitor cell; DMP: differentially methylated probe; GMP: granulocyte-macrophage progenitor cell; kd: knockdown; LDMNC: low density mononuclear cell; MCIp: methyl-CpG immunoprecipitation; MDS: myelodysplastic syndrome; MEP: megakaryocyte-erythroid progenitor cell; PCA: principal component analysis; RA: refractory anemia; RAEB: refractory anemia with excess of blasts; RARS: refractory anemia with ringed sideroblasts; RCMD: refractory cytopenia with multilineage dysplasia; RCMD-RS: refractory cytopenia with multilineage dysplasia and ringed sideroblasts; SAM: significance analysis of microarrays; WHO: World Health Organization; wt: wild type.

## Competing interests

The authors declare that they have no competing interests.

## Authors’ contributions

MS collected mouse specimens, conducted experiments and wrote the manuscript. RC was responsible for conception of the project as well as for experimental planning, preparation, and writing of the manuscript. NB and MZ performed statistical analyses. JP performed mouse breeding and collection of mouse specimens. DBL, CCO and LB contributed to the conception of the experimental setup. LG performed motif search and bioinformatics analysis of array data. MA, S-ZK-W and PS contributed to histological examinations of mouse specimens. TW isolated DNA from CD34+ cells and healthy granulocytes and performed MassARRAY analysis on CD34+ cell DNA. AL and MDM performed cell sorting. MW and NG collected and provided MDS patient samples. ML collected and provided human AML samples. FR provided the PU.1 animals. MR contributed assay development and data analyses. DW was involved in experimental conception and conduction and writing of the manuscript. CP contributed to experimental planning and preparation of the manuscript. All authors read, reviewed and finally approved the manuscript.

## Authors’ information

MS and TW hold a stipend of the Helmholtz International Graduate School.

## Supplementary Material

Additional file 1 A table listing the primers used for MassARRAY analysis. Click here for file

Additional file 2 **A circos plot displaying the hypomethylated probes in PU.1-kd animals.** The outer circle represents the different G-banded mouse chromosomes, lines in the inner circles depict significantly hypomethylated probes of the different disease stages (preleukemic, early leukemic and late leukemic from inner to outer). Click here for file

Additional file 3 **A table listing the aberrantly methylated probes of the preleukemic stage.** The table shows probe ID (column A), chromosome (B), start (C), end (D), the genomic location (E), the corresponding gene name (F), the location of the respective probe (G to J), the methylation status in the preleukemic stage (K) and the methylation status in the three disease stages (L,M). Click here for file

Additional file 4 **A figure displaying the validation of array-based screening results by quantitative DNA methylation analysis (MassARRAY).** Forty preleukemic hypermethylated genes were randomly selected for MassARRAY analysis (see also Figure 2 for *Fzd5* and *Fzd8*). Dotplots depict average methylation per amplicon and sample; the median of a sample group is represented by a black bar. Mann-Whitney U test was used to test for differences between wild type and the different disease stages and also between the disease stages (*P < 0.05, **P < 0.01, ***P ≤ 0.001). Click here for file

Additional file 5 **(A-H) The MassARRAY results for *Prdm16 *(A), *Robo3 *(B), *Bcor *(C,D), *Hes6 *(E), *Tal1 *(F) and *Itpka *(G,H) in sorted cells from preleukemic PU.1-kd mice and PU.1-wt animals.** The sorted cells include LSKs (lineage-negative, Ska1-positive, c-kit negative cells), CMPs, GMPs and MEPs. Click here for file

Additional file 6 **A table showing the comparison between aberrantly methylated genes of the preleukemic stage and previously published genome-wide DNA methylation data.** The two tables show the overlap of genes of the preleukemic stage with publicly available genome-wide methylation data in AML (left table) and MDS (right table). Columns B and I indicate if the gene was hypermethylated in the promoter region of the preleukemic hypermethylated genes. Click here for file

Additional file 7 **A table listing pathways that are enriched in the aberrantly methylated genes of the preleukemic stage.** Column A, respective pathway; column B, log *P*-value; column C, enrichment score; column D, gene names. Click here for file

Additional file 8 **A figure showing the DNA methylation of *FZD5 *and *FZD8 *in different risk groups of MDS patients.** DNA methylation in WHO groups (5q-, deletion of chromosome 5q; RA, refractory anemia; RARS, refractory anemia with ringed sideroblasts; RCMD, refractory cytopenia with multilineage dysplasia; RCMD-RS, refractory cytopenia with multilineade dysplasia and ringed sideroblasts; RAEB, refractory anemia with excess of blasts; CMML, chronic myelo-monocytic leukemia) or IPSS (International Prognostic Scoring System) classification risk groups (low, Int1, Int2, high) compared to healthy granulocytes are shown. Mann-Whitney U test was used to test for differences between the risk groups/WHO classification groups and healthy granulocytes (*P < 0.05, **P < 0.01, ***P ≤ 0.001). Click here for file

Additional file 9 **A figure depicting the quantitative determination of DNA methylation in human AML samples.** (A-C) Heatmaps (left) and dotplots (right) of amplicons from *PRDM16* (A), *ROBO3* (B) and *CXCL14* (C) in AML patients, healthy granulocytes, and in CD34+ cells are shown. Heatmaps display single CpG units (columns) of AML patients, healthy granulocytes and CD34+ cells. Differently colored bars to the left of the heatmaps indicate AML (A, red), healthy granulocytes (G, green) and CD34+ cells (C, grey). Methylation values range from 0% (light green) to 100% (dark blue). Schemes above the heatmaps display the gene (black bar), transcription start (arrow), the relative location of the CpG islands (CGI) and the analyzed amplicons (MA). Dotplots show average methylation per amplicon of AML patients and of healthy granulocytes (HG). Median methylation of a sample group (median methylation in AML samples for PRDM16, 5%; for ROBO3, 7.5%; and for CXCL14, 17.3%) is depicted by a black bar. Mann-Whitney U test was used to test for differences between AML samples and healthy granulocytes/CD34+ cells (*P < 0.05, **P <0.01, ***P < 0.001). Click here for file

Additional file 10 **A table showing overrepresented motifs in hypermethylated CGIs of the preleukemic stage.** The table shows the results of *de novo* motif search of the preleukemic hypermethylated CGIs compared to whole genome background enrichment (47,511 target sequences). Click here for file

Additional file 11 **A table showing the overlap of preleukemic hypermethylated CGIs (covered by at least two hypermethylated probes) with genomic coordinates of PU.1 ChIP-Seq data.** The table shows chromosomal location of the respective gene (columns A to D) and the gene name (column E) of overlapping preleukemic hypermethylated CGIs and publicly available ChIP-Seq data (selected by the more stringent criteria of two hypermethylated probes covering the gene). Click here for file

Additional file 12 **A figure depicting the quantitative determination of DNA methylation in PU.1 target genes. (A-C)** Heatmaps (left) and dotplots (right) of amplicons from *Hes6* (A), *Tal1* (B) and *Itpka* (C) in PU.1-wt animals and different disease stages are shown. Heatmaps display single CpG units (columns) of different PU.1-wt and PU.1-kd animals. Differently colored bars to the right of the heatmaps indicate preleukemic (P, black), early leukemic (EL, blue), late leukemic (LL, red), and PU.1-wt (W, green). *In vitro* methylated standard DNA (0%, 20%, 40%, 60%, 80% and 100% DNA methylation; S, grey) served as control. Methylation values range from 0% (light green) to 100% (dark blue). Schemes above the heatmaps display the gene (black bar), transcription start (arrow), the PU.1 ChIP peak (PU1), the relative location of the CpG islands (CGI) and the analyzed amplicons (MA). Dotplots show average methylation per amplicon of PU.1-wt animals and the different disease stages. Median methylation of a sample group is depicted by a black bar. Mann-Whitney U test was used to test for differences between PU.1-wt and stages and within the different stages (*P < 0.05, **P <0.01, ***P < 0.001). Click here for file
