# Supplementary material for: Early aberrant DNA methylation events in a mouse model of acute myeloid leukemia
Source: Genome Med. 2014 Apr 30;6(4):34. doi: 10.1186/gm551 (PMC4062060; doi:10.1186/gm551)
Supplement: Additional file 2 — A circos plot displaying the hypomethylated probes in PU.1-kd animals. The outer circle represents the different G-banded mouse chromosomes, lines in the inner circles depict significantly hypomethylated probes of the different disease stages (preleukemic, early leukemic and late leukemic from inner to outer). [file gm551-S2.pptx]

## Slide 1
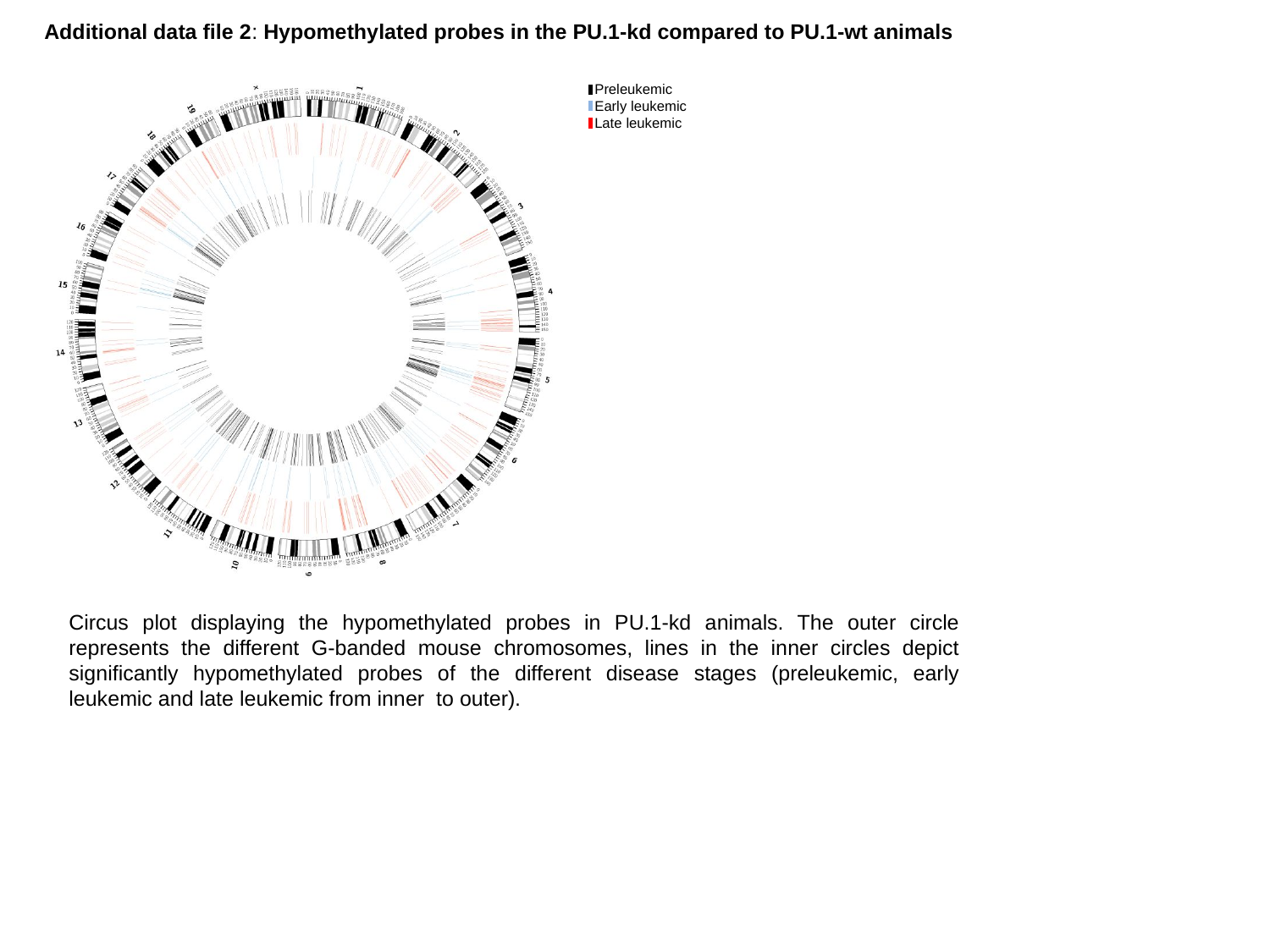

Additional data file 2: Hypomethylated probes in the PU.1-kd compared to PU.1-wt animals
Preleukemic
Early leukemic
Late leukemic
Circus plot displaying the hypomethylated probes in PU.1-kd animals. The outer circle represents the different G-banded mouse chromosomes, lines in the inner circles depict significantly hypomethylated probes of the different disease stages (preleukemic, early leukemic and late leukemic from inner to outer).
