# Supplementary material for: Early aberrant DNA methylation events in a mouse model of acute myeloid leukemia
Source: Genome Med. 2014 Apr 30;6(4):34. doi: 10.1186/gm551 (PMC4062060; doi:10.1186/gm551)
Supplement: Additional file 4 — A figure displaying the validation of array-based screening results by quantitative DNA methylation analysis (MassARRAY). Forty preleukemic hypermethylated genes were randomly selected for MassARRAY analysis (see also Figure 2 for Fzd5 and Fzd8). Dotplots depict average methylation per amplicon and sample; the median of a sample group is represented by a black bar. Mann-Whitney U test was used to test for differences between wild type and the different disease stages and also between the disease stages (*P < 0.05, **P < 0.01, ***P ≤ 0.001). [file gm551-S4.pptx]

## Slide 1
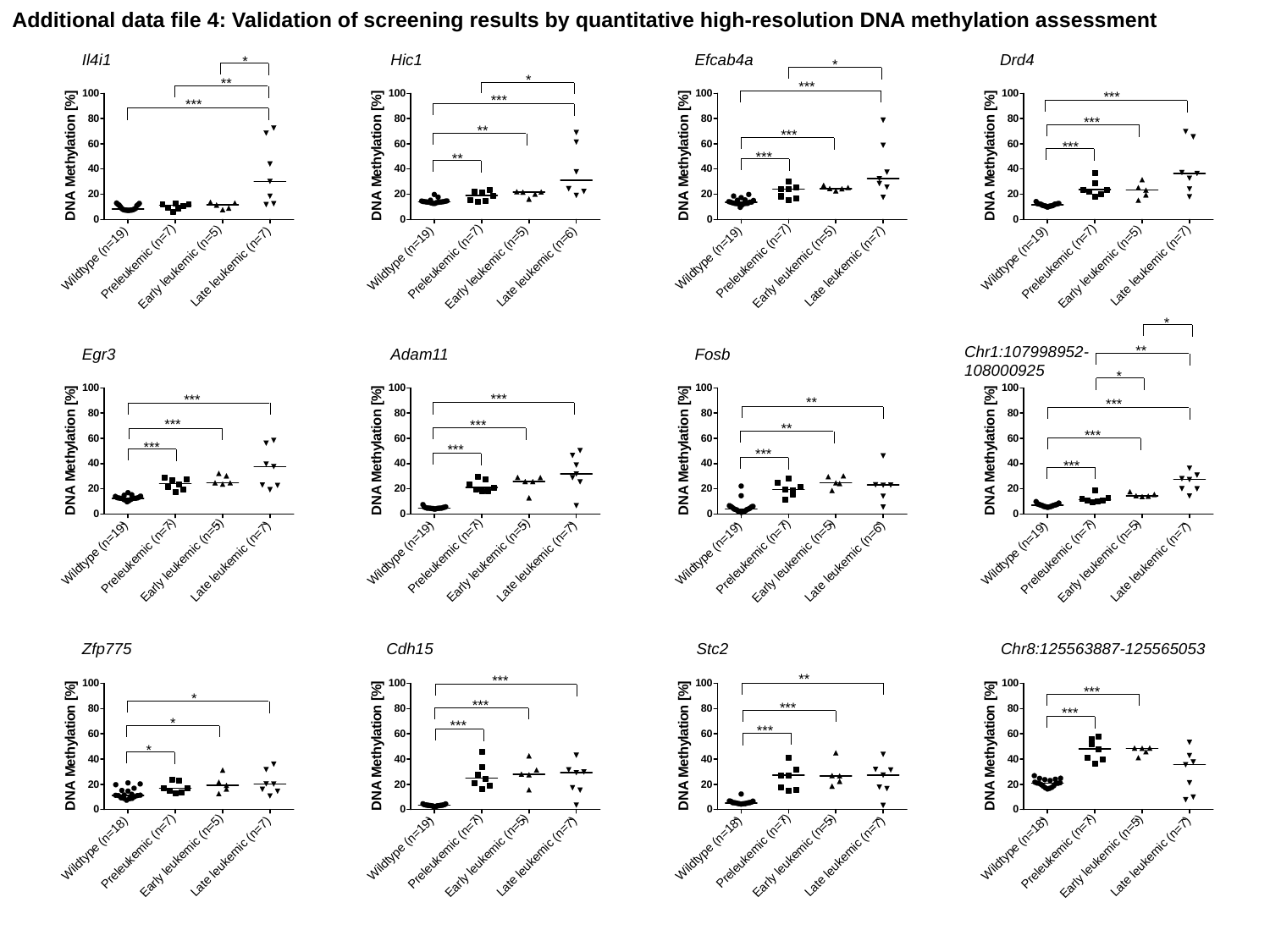

Additional data file 4: Validation of screening results by quantitative high-resolution DNA methylation assessment
Il4i1
Hic1
Efcab4a
Drd4
*
*
*
**
***
***
***
***
***
**
***
***
***
**
Wildtype (n=19)
Preleukemic (n=7)
Late leukemic (n=7)
Early leukemic (n=5)
Wildtype (n=19)
Preleukemic (n=7)
Late leukemic (n=7)
Early leukemic (n=5)
Wildtype (n=19)
Preleukemic (n=7)
Late leukemic (n=7)
Early leukemic (n=5)
Wildtype (n=19)
Preleukemic (n=7)
Late leukemic (n=6)
Early leukemic (n=5)
*
Chr1:107998952-108000925
**
Egr3
Adam11
Fosb
*
***
***
**
***
***
***
**
***
***
***
***
***
Wildtype (n=19)
Preleukemic (n=7)
Early leukemic (n=5)
Late leukemic (n=7)
Wildtype (n=19)
Preleukemic (n=7)
Early leukemic (n=5)
Late leukemic (n=7)
Wildtype (n=19)
Preleukemic (n=7)
Early leukemic (n=5)
Late leukemic (n=6)
Wildtype (n=19)
Preleukemic (n=7)
Early leukemic (n=5)
Late leukemic (n=7)
Zfp775
Cdh15
Stc2
Chr8:125563887-125565053
**
***
***
*
***
***
***
*
***
***
*
Wildtype (n=18)
Preleukemic (n=7)
Early leukemic (n=5)
Late leukemic (n=7)
Wildtype (n=19)
Preleukemic (n=7)
Early leukemic (n=5)
Late leukemic (n=7)
Wildtype (n=18)
Preleukemic (n=7)
Early leukemic (n=5)
Late leukemic (n=7)
Wildtype (n=18)
Preleukemic (n=7)
Late leukemic (n=7)
Early leukemic (n=5)

## Slide 2
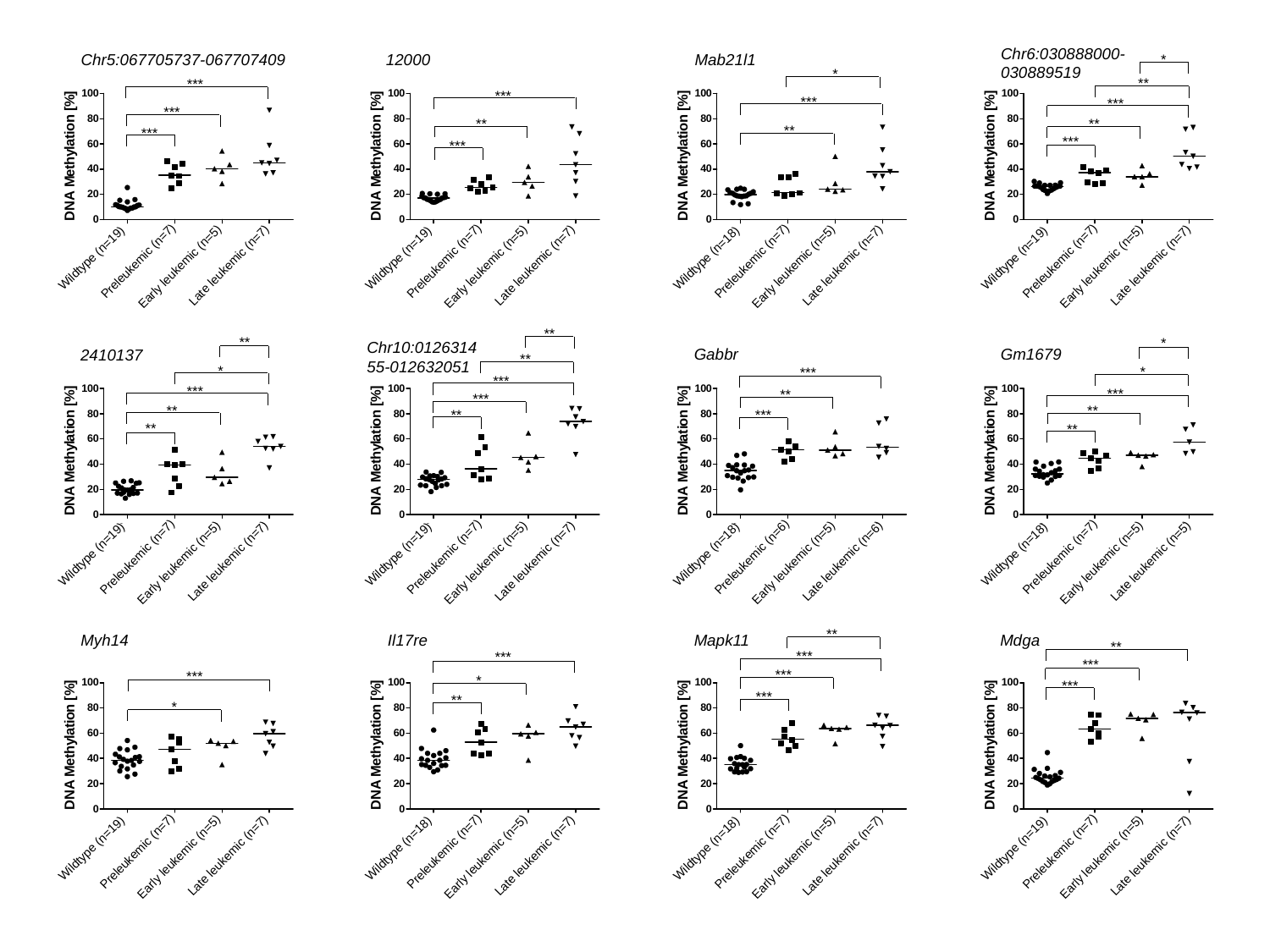

Chr6:030888000-030889519
Chr5:067705737-067707409
12000
Mab21l1
*
*
**
***
***
***
***
***
**
**
**
***
***
***
Wildtype (n=19)
Preleukemic (n=7)
Late leukemic (n=7)
Early leukemic (n=5)
Wildtype (n=19)
Preleukemic (n=7)
Late leukemic (n=7)
Early leukemic (n=5)
Wildtype (n=18)
Preleukemic (n=7)
Late leukemic (n=7)
Early leukemic (n=5)
Wildtype (n=19)
Preleukemic (n=7)
Late leukemic (n=7)
Early leukemic (n=5)
**
**
*
Chr10:012631455-012632051
Gabbr
Gm1679
2410137
**
*
*
***
***
***
***
**
***
**
**
**
***
**
**
Wildtype (n=19)
Preleukemic (n=7)
Late leukemic (n=7)
Early leukemic (n=5)
Wildtype (n=19)
Preleukemic (n=7)
Late leukemic (n=7)
Early leukemic (n=5)
Wildtype (n=18)
Preleukemic (n=6)
Late leukemic (n=6)
Early leukemic (n=5)
Wildtype (n=18)
Preleukemic (n=7)
Late leukemic (n=5)
Early leukemic (n=5)
**
Myh14
Il17re
Mapk11
Mdga
**
***
***
***
***
***
*
***
***
**
*
Wildtype (n=19)
Preleukemic (n=7)
Late leukemic (n=7)
Early leukemic (n=5)
Wildtype (n=18)
Preleukemic (n=7)
Late leukemic (n=7)
Early leukemic (n=5)
Wildtype (n=18)
Preleukemic (n=7)
Late leukemic (n=7)
Early leukemic (n=5)
Wildtype (n=19)
Preleukemic (n=7)
Late leukemic (n=7)
Early leukemic (n=5)

## Slide 3
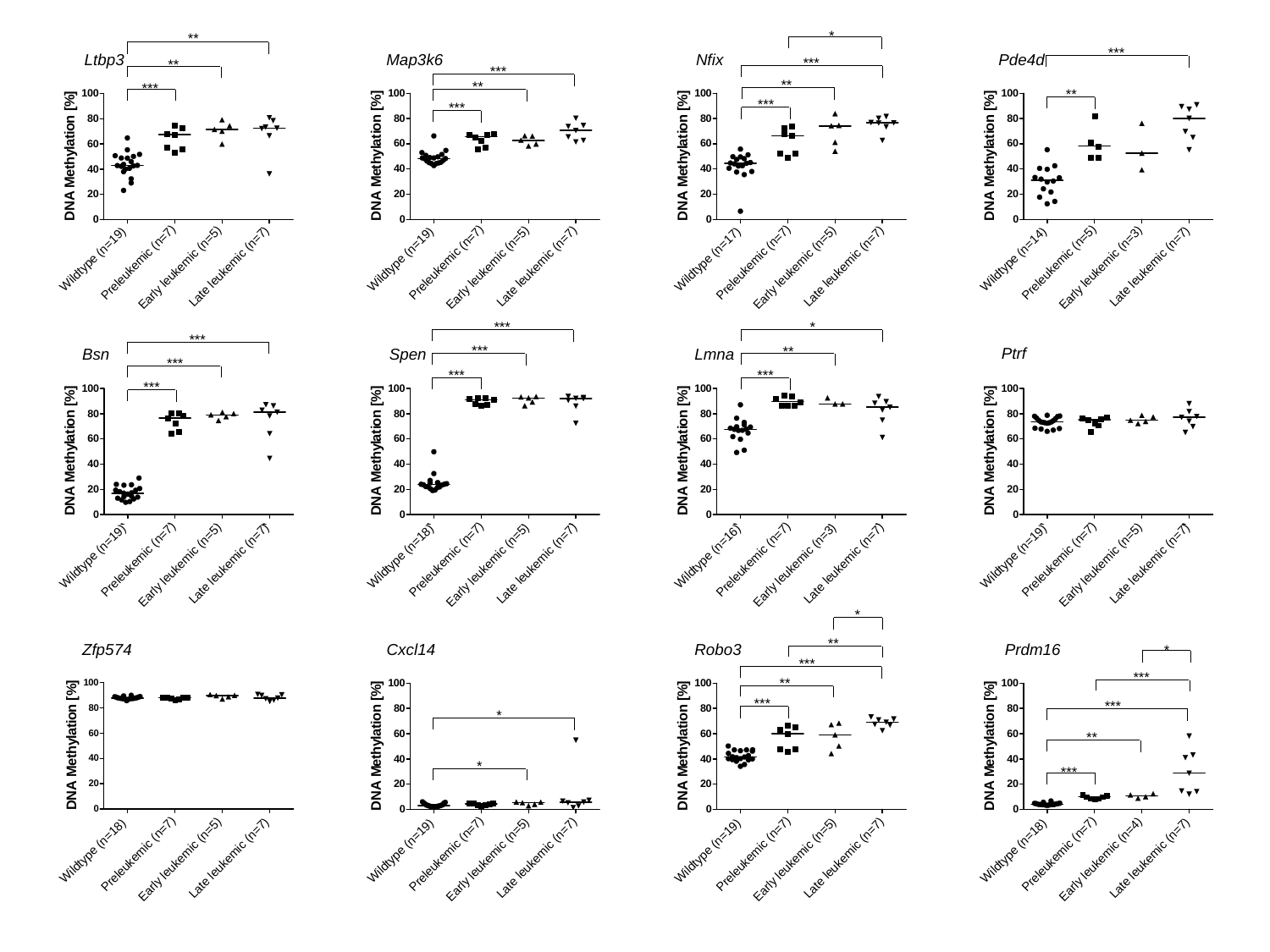

*
**
***
Ltbp3
Map3k6
Nfix
Pde4d
***
**
***
**
**
***
**
***
***
Wildtype (n=19)
Preleukemic (n=7)
Late leukemic (n=7)
Early leukemic (n=5)
Wildtype (n=19)
Preleukemic (n=7)
Late leukemic (n=7)
Early leukemic (n=5)
Wildtype (n=17)
Preleukemic (n=7)
Late leukemic (n=7)
Early leukemic (n=5)
Wildtype (n=14)
Preleukemic (n=5)
Late leukemic (n=7)
Early leukemic (n=3)
***
*
***
***
**
Ptrf
Bsn
Spen
Lmna
***
***
***
***
Wildtype (n=19)
Preleukemic (n=7)
Late leukemic (n=7)
Early leukemic (n=5)
Wildtype (n=18)
Preleukemic (n=7)
Late leukemic (n=7)
Early leukemic (n=5)
Wildtype (n=16)
Preleukemic (n=7)
Late leukemic (n=7)
Early leukemic (n=3)
Wildtype (n=19)
Preleukemic (n=7)
Late leukemic (n=7)
Early leukemic (n=5)
*
**
Zfp574
Cxcl14
Robo3
Prdm16
*
***
***
**
***
***
*
**
 *
***
Wildtype (n=18)
Preleukemic (n=7)
Late leukemic (n=7)
Early leukemic (n=5)
Wildtype (n=19)
Preleukemic (n=7)
Late leukemic (n=7)
Early leukemic (n=5)
Wildtype (n=19)
Preleukemic (n=7)
Late leukemic (n=7)
Early leukemic (n=5)
Wildtype (n=18)
Preleukemic (n=7)
Late leukemic (n=7)
Early leukemic (n=4)

## Slide 4
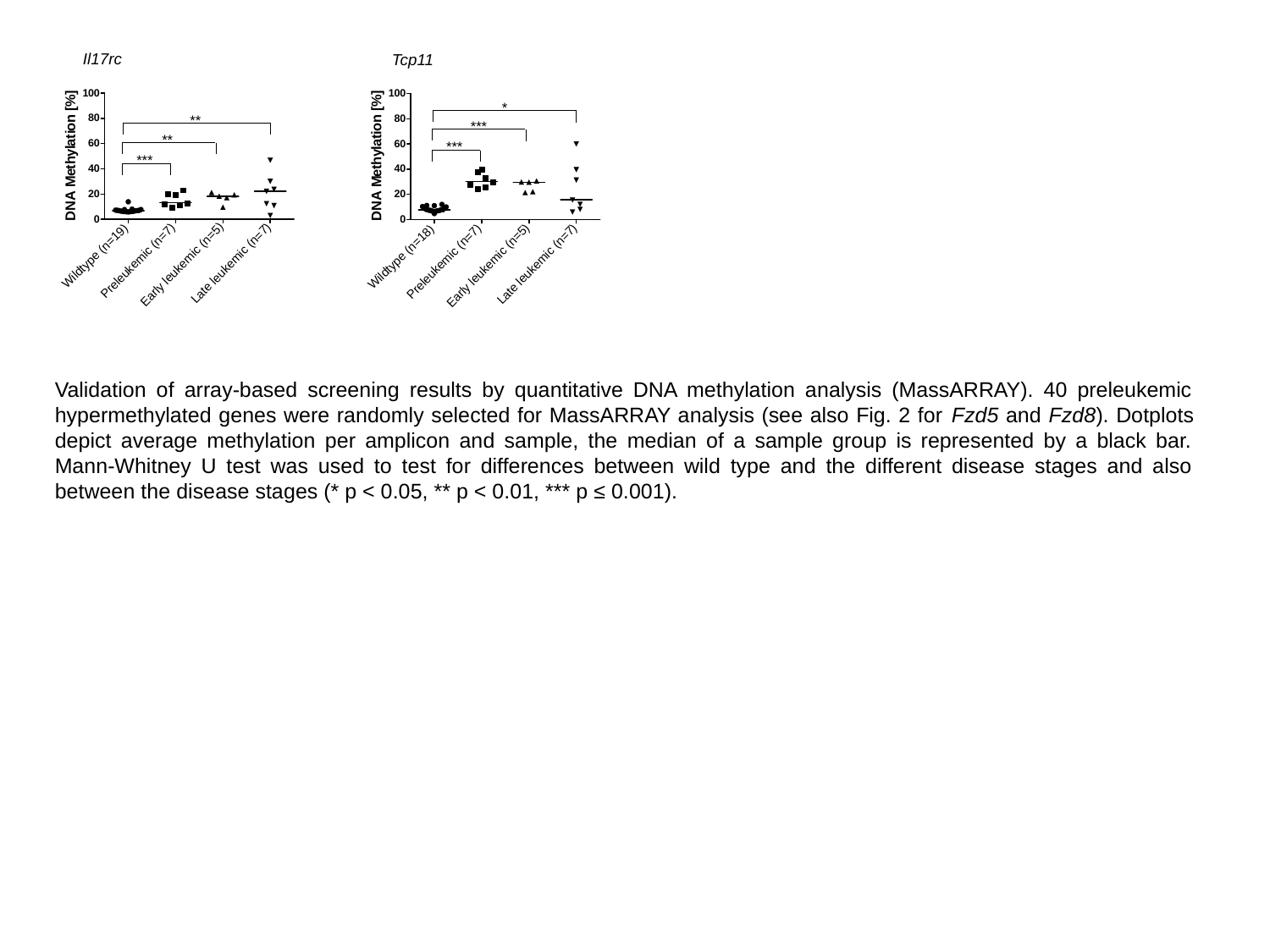

Il17rc
Tcp11
*
**
***
**
***
***
Wildtype (n=19)
Preleukemic (n=7)
Late leukemic (n=7)
Early leukemic (n=5)
Wildtype (n=18)
Preleukemic (n=7)
Late leukemic (n=7)
Early leukemic (n=5)
Validation of array-based screening results by quantitative DNA methylation analysis (MassARRAY). 40 preleukemic hypermethylated genes were randomly selected for MassARRAY analysis (see also Fig. 2 for Fzd5 and Fzd8). Dotplots depict average methylation per amplicon and sample, the median of a sample group is represented by a black bar. Mann-Whitney U test was used to test for differences between wild type and the different disease stages and also between the disease stages (* p < 0.05, ** p < 0.01, *** p ≤ 0.001).
