# Supplementary material for: Early aberrant DNA methylation events in a mouse model of acute myeloid leukemia
Source: Genome Med. 2014 Apr 30;6(4):34. doi: 10.1186/gm551 (PMC4062060; doi:10.1186/gm551)
Supplement: Additional file 5 — (A-H) The MassARRAY results for Prdm16 (A), Robo3 (B), Bcor (C,D), Hes6 (E), Tal1 (F) and Itpka (G,H) in sorted cells from preleukemic PU.1-kd mice and PU.1-wt animals. The sorted cells include LSKs (lineage-negative, Ska1-positive, c-kit negative cells), CMPs, GMPs and MEPs. [file gm551-S5.pptx]

## Slide 1
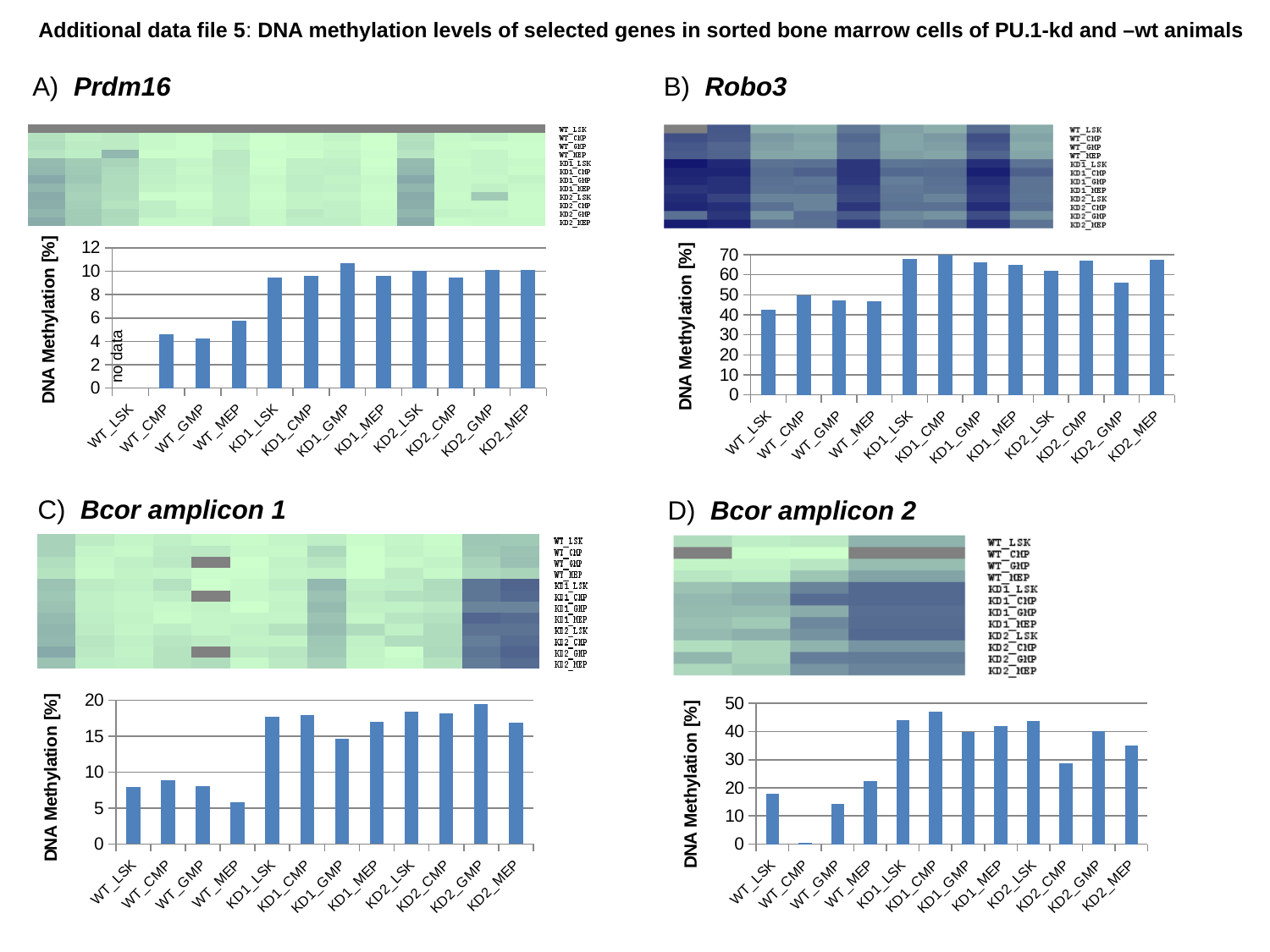

Additional data file 5: DNA methylation levels of selected genes in sorted bone marrow cells of PU.1-kd and –wt animals
A) Prdm16
B) Robo3
### Chart
| Category | |
|---|---|
| WT_LSK | 0.0 |
| WT_CMP | 4.572857142857144 |
| WT_GMP | 4.215714285714286 |
| WT_MEP | 5.785714285714287 |
| KD1_LSK | 9.430000000000001 |
| KD1_CMP | 9.571428571428573 |
| KD1_GMP | 10.714285714285717 |
| KD1_MEP | 9.642857142857144 |
| KD2_LSK | 10.071428571428573 |
| KD2_CMP | 9.42857142857143 |
| KD2_GMP | 10.142857142857144 |
| KD2_MEP | 10.142857142857146 |
### Chart
| Category | |
|---|---|
| WT_LSK | 42.75 |
| WT_CMP | 49.888888888888886 |
| WT_GMP | 47.333333333333336 |
| WT_MEP | 46.666666666666664 |
| KD1_LSK | 67.88888888888889 |
| KD1_CMP | 70.88888888888889 |
| KD1_GMP | 66.33333333333333 |
| KD1_MEP | 65.0 |
| KD2_LSK | 62.11111111111111 |
| KD2_CMP | 67.00000000000001 |
| KD2_GMP | 56.00000000000001 |
| KD2_MEP | 67.44444444444444 |no data
C) Bcor amplicon 1
D) Bcor amplicon 2
### Chart
| Category | |
|---|---|
| WT_LSK | 7.923076923076923 |
| WT_CMP | 8.923846153846155 |
| WT_GMP | 8.084999999999999 |
| WT_MEP | 5.846923076923078 |
| KD1_LSK | 17.693076923076923 |
| KD1_CMP | 17.999999999999996 |
| KD1_GMP | 14.693076923076923 |
| KD1_MEP | 17.0 |
| KD2_LSK | 18.384615384615387 |
| KD2_CMP | 18.153846153846153 |
| KD2_GMP | 19.499999999999996 |
| KD2_MEP | 16.923076923076923 |
### Chart
| Category | |
|---|---|
| WT_LSK | 18.000000000000004 |
| WT_CMP | 0.505 |
| WT_GMP | 14.399999999999999 |
| WT_MEP | 22.400000000000002 |
| KD1_LSK | 43.99999999999999 |
| KD1_CMP | 46.99999999999999 |
| KD1_GMP | 40.0 |
| KD1_MEP | 41.8 |
| KD2_LSK | 43.8 |
| KD2_CMP | 28.799999999999997 |
| KD2_GMP | 40.199999999999996 |
| KD2_MEP | 35.0 |

## Slide 2
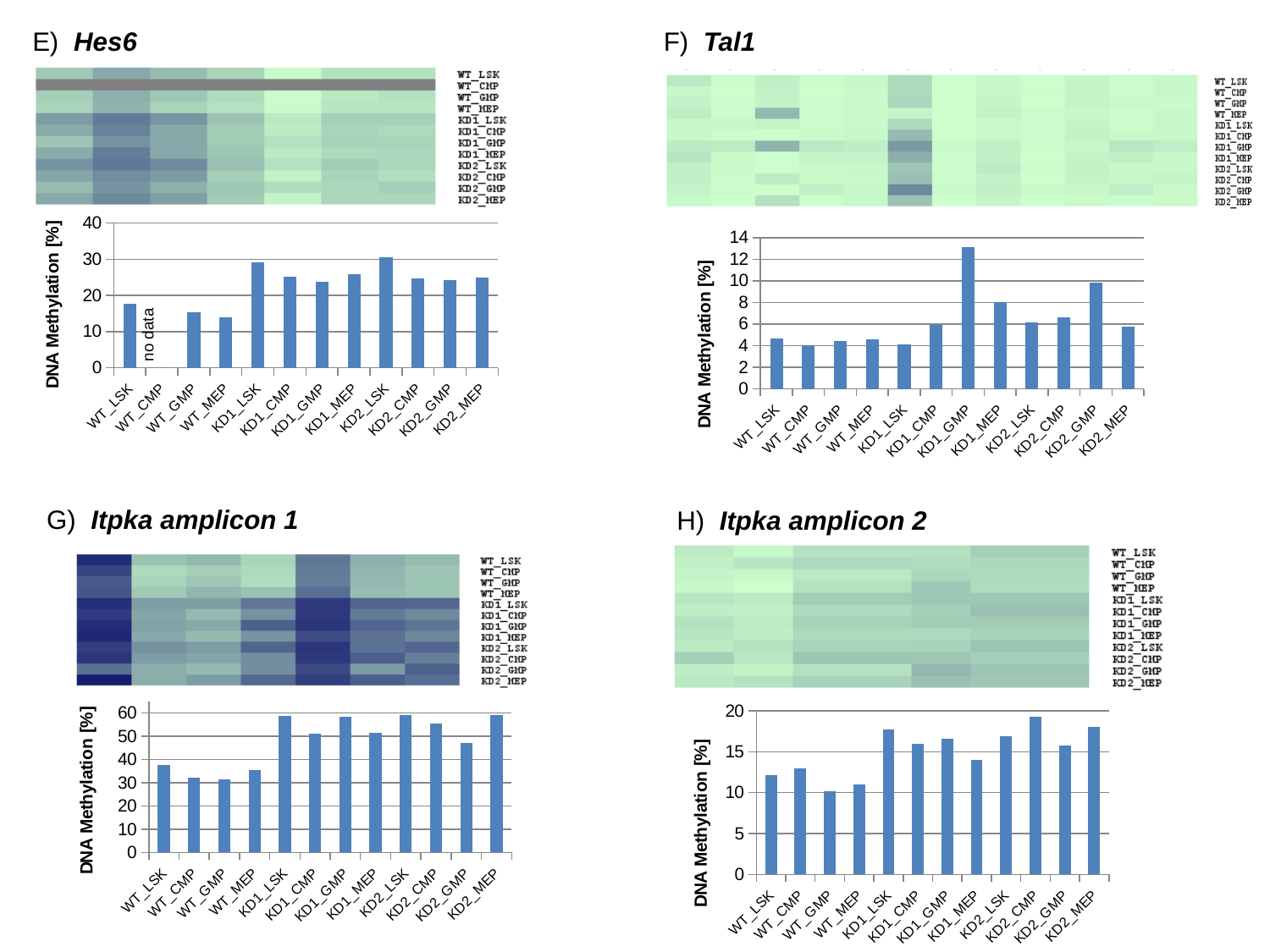

E) Hes6
F) Tal1
### Chart
| Category | |
|---|---|
| WT_LSK | 17.71428571428572 |
| WT_CMP | 0.0 |
| WT_GMP | 15.42857142857143 |
| WT_MEP | 13.999999999999998 |
| KD1_LSK | 29.142857142857142 |
| KD1_CMP | 25.142857142857146 |
| KD1_GMP | 23.85714285714285 |
| KD1_MEP | 25.857142857142858 |
| KD2_LSK | 30.571428571428566 |
| KD2_CMP | 24.714285714285715 |
| KD2_GMP | 24.14285714285714 |
| KD2_MEP | 25.0 |
### Chart
| Category | |
|---|---|
| WT_LSK | 4.693846153846155 |
| WT_CMP | 4.002307692307692 |
| WT_GMP | 4.463076923076924 |
| WT_MEP | 4.616153846153847 |
| KD1_LSK | 4.1546153846153855 |
| KD1_CMP | 5.924615384615385 |
| KD1_GMP | 13.153846153846155 |
| KD1_MEP | 8.001538461538463 |
| KD2_LSK | 6.154615384615385 |
| KD2_CMP | 6.616153846153846 |
| KD2_GMP | 9.846923076923078 |
| KD2_MEP | 5.772307692307693 |no data
G) Itpka amplicon 1
H) Itpka amplicon 2
### Chart
| Category | |
|---|---|
| WT_LSK | 12.14285714285714 |
| WT_CMP | 13.0 |
| WT_GMP | 10.142857142857144 |
| WT_MEP | 11.001428571428571 |
| KD1_LSK | 17.71428571428571 |
| KD1_CMP | 16.0 |
| KD1_GMP | 16.57142857142857 |
| KD1_MEP | 13.999999999999998 |
| KD2_LSK | 16.857142857142858 |
| KD2_CMP | 19.285714285714285 |
| KD2_GMP | 15.714285714285717 |
| KD2_MEP | 18.0 |
### Chart
| Category | |
|---|---|
| WT_LSK | 37.57142857142857 |
| WT_CMP | 32.285714285714285 |
| WT_GMP | 31.285714285714285 |
| WT_MEP | 35.285714285714285 |
| KD1_LSK | 58.57142857142858 |
| KD1_CMP | 51.142857142857146 |
| KD1_GMP | 58.285714285714285 |
| KD1_MEP | 51.42857142857142 |
| KD2_LSK | 59.0 |
| KD2_CMP | 55.57142857142857 |
| KD2_GMP | 47.285714285714285 |
| KD2_MEP | 59.285714285714285 |

## Slide 3
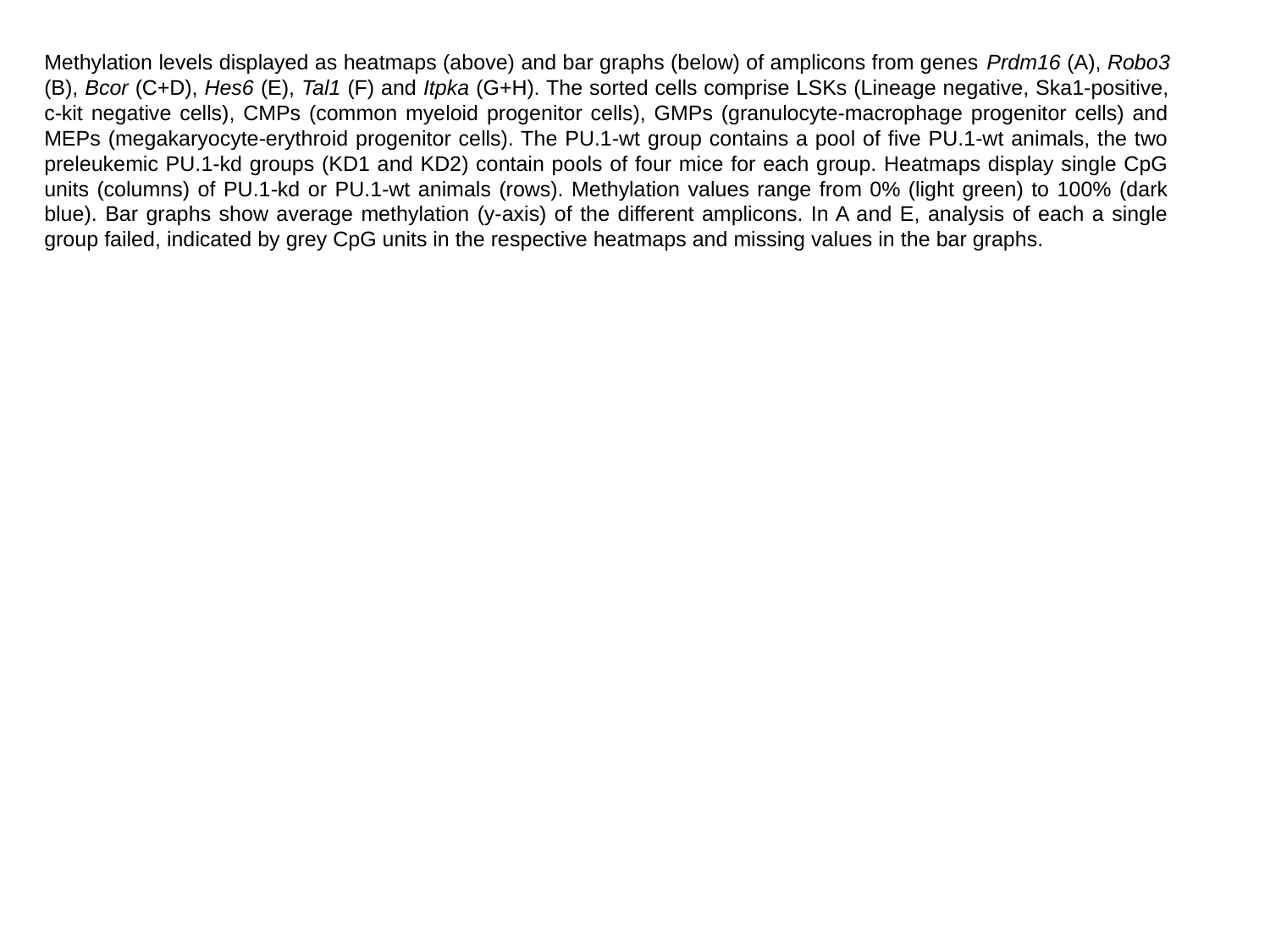

Methylation levels displayed as heatmaps (above) and bar graphs (below) of amplicons from genes Prdm16 (A), Robo3 (B), Bcor (C+D), Hes6 (E), Tal1 (F) and Itpka (G+H). The sorted cells comprise LSKs (Lineage negative, Ska1-positive, c-kit negative cells), CMPs (common myeloid progenitor cells), GMPs (granulocyte-macrophage progenitor cells) and MEPs (megakaryocyte-erythroid progenitor cells). The PU.1-wt group contains a pool of five PU.1-wt animals, the two preleukemic PU.1-kd groups (KD1 and KD2) contain pools of four mice for each group. Heatmaps display single CpG units (columns) of PU.1-kd or PU.1-wt animals (rows). Methylation values range from 0% (light green) to 100% (dark blue). Bar graphs show average methylation (y-axis) of the different amplicons. In A and E, analysis of each a single group failed, indicated by grey CpG units in the respective heatmaps and missing values in the bar graphs.
