# Supplementary material for: Early aberrant DNA methylation events in a mouse model of acute myeloid leukemia
Source: Genome Med. 2014 Apr 30;6(4):34. doi: 10.1186/gm551 (PMC4062060; doi:10.1186/gm551)
Supplement: Additional file 8 — A figure showing the DNA methylation of FZD5 and FZD8 in different risk groups of MDS patients. DNA methylation in WHO groups (5q-, deletion of chromosome 5q; RA, refractory anemia; RARS, refractory anemia with ringed sideroblasts; RCMD, refractory cytopenia with multilineage dysplasia; RCMD-RS, refractory cytopenia with multilineade dysplasia and ringed sideroblasts; RAEB, refractory anemia with excess of blasts; CMML, chronic myelo-monocytic leukemia) or IPSS (International Prognostic Scoring System) classification risk groups (low, Int1, Int2, high) compared to healthy granulocytes are shown. Mann-Whitney U test was used to test for differences between the risk groups/WHO classification groups and healthy granulocytes (*P < 0.05, **P < 0.01, ***P ≤ 0.001). [file gm551-S8.pptx]

## Slide 1
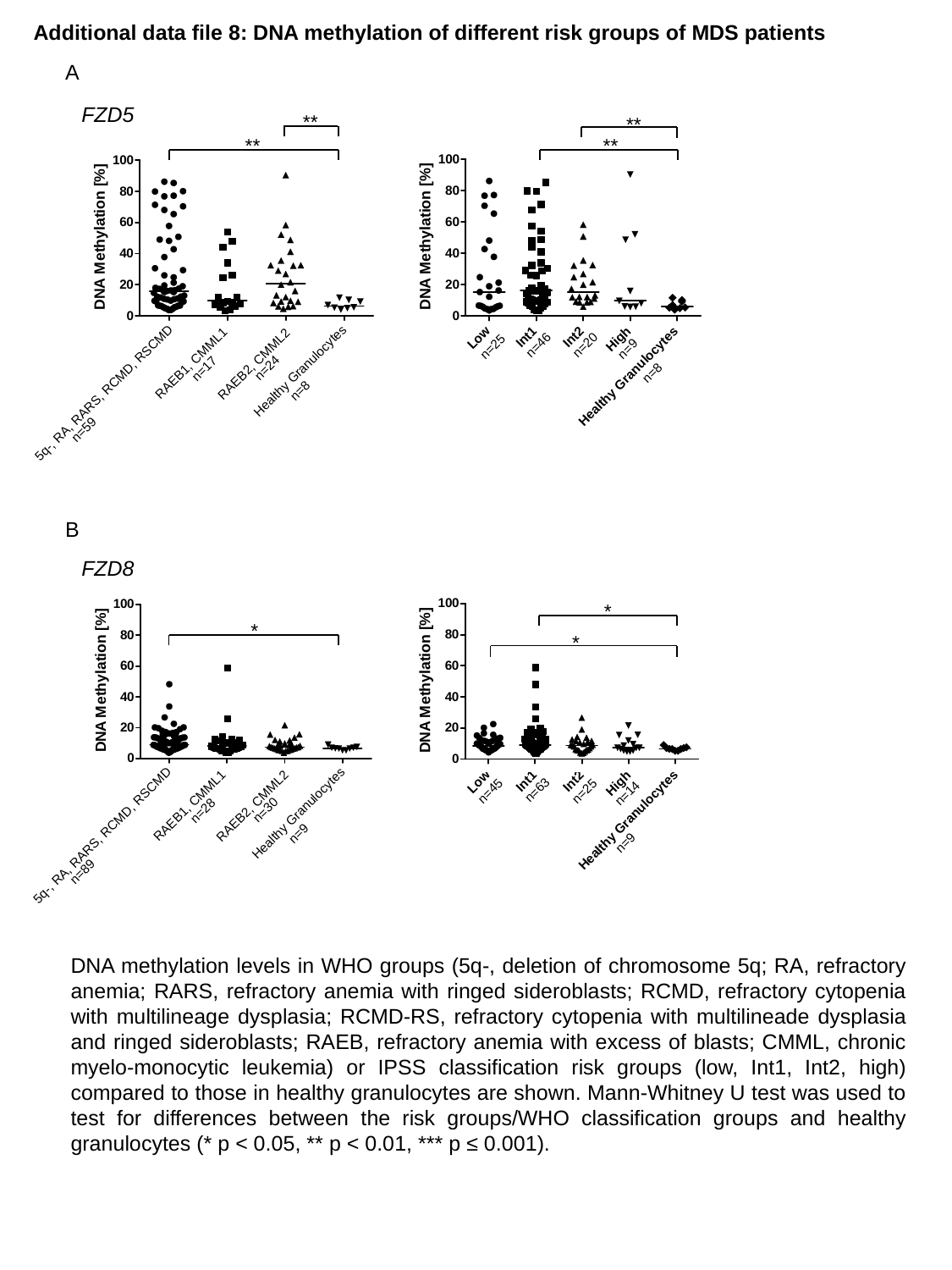

Additional data file 8: DNA methylation of different risk groups of MDS patients
A
FZD5
**
**
**
**
n=20
n=46
n=9
n=25
n=8
RAEB1, CMML1
n=17
n=24
5q-, RA, RARS, RCMD, RSCMD
n=59
n=8
Healthy Granulocytes
RAEB2, CMML2
B
FZD8
*
*
*
n=63
n=45
n=25
n=14
RAEB1, CMML1
RAEB2, CMML2
Healthy Granulocytes
n=30
n=28
5q-, RA, RARS, RCMD, RSCMD
n=9
n=89
n=9
DNA methylation levels in WHO groups (5q-, deletion of chromosome 5q; RA, refractory anemia; RARS, refractory anemia with ringed sideroblasts; RCMD, refractory cytopenia with multilineage dysplasia; RCMD-RS, refractory cytopenia with multilineade dysplasia and ringed sideroblasts; RAEB, refractory anemia with excess of blasts; CMML, chronic myelo-monocytic leukemia) or IPSS classification risk groups (low, Int1, Int2, high) compared to those in healthy granulocytes are shown. Mann-Whitney U test was used to test for differences between the risk groups/WHO classification groups and healthy granulocytes (* p < 0.05, ** p < 0.01, *** p ≤ 0.001).
