# Supplementary material for: Early aberrant DNA methylation events in a mouse model of acute myeloid leukemia
Source: Genome Med. 2014 Apr 30;6(4):34. doi: 10.1186/gm551 (PMC4062060; doi:10.1186/gm551)
Supplement: Additional file 9 — A figure depicting the quantitative determination of DNA methylation in human AML samples. (A-C) Heatmaps (left) and dotplots (right) of amplicons from PRDM16 (A), ROBO3 (B) and CXCL14 (C) in AML patients, healthy granulocytes, and in CD34+ cells are shown. Heatmaps display single CpG units (columns) of AML patients, healthy granulocytes and CD34+ cells. Differently colored bars to the left of the heatmaps indicate AML (A, red), healthy granulocytes (G, green) and CD34+ cells (C, grey). Methylation values range from 0% (light green) to 100% (dark blue). Schemes above the heatmaps display the gene (black bar), transcription start (arrow), the relative location of the CpG islands (CGI) and the analyzed amplicons (MA). Dotplots show average methylation per amplicon of AML patients and of healthy granulocytes (HG). Median methylation of a sample group (median methylation in AML samples for PRDM16, 5%; for ROBO3, 7.5%; and for CXCL14, 17.3%) is depicted by a black bar. Mann-Whitney U test was used to test for differences between AML samples and healthy granulocytes/CD34+ cells (*P < 0.05, **P <0.01, ***P < 0.001). [file gm551-S9.pptx]

## Slide 1
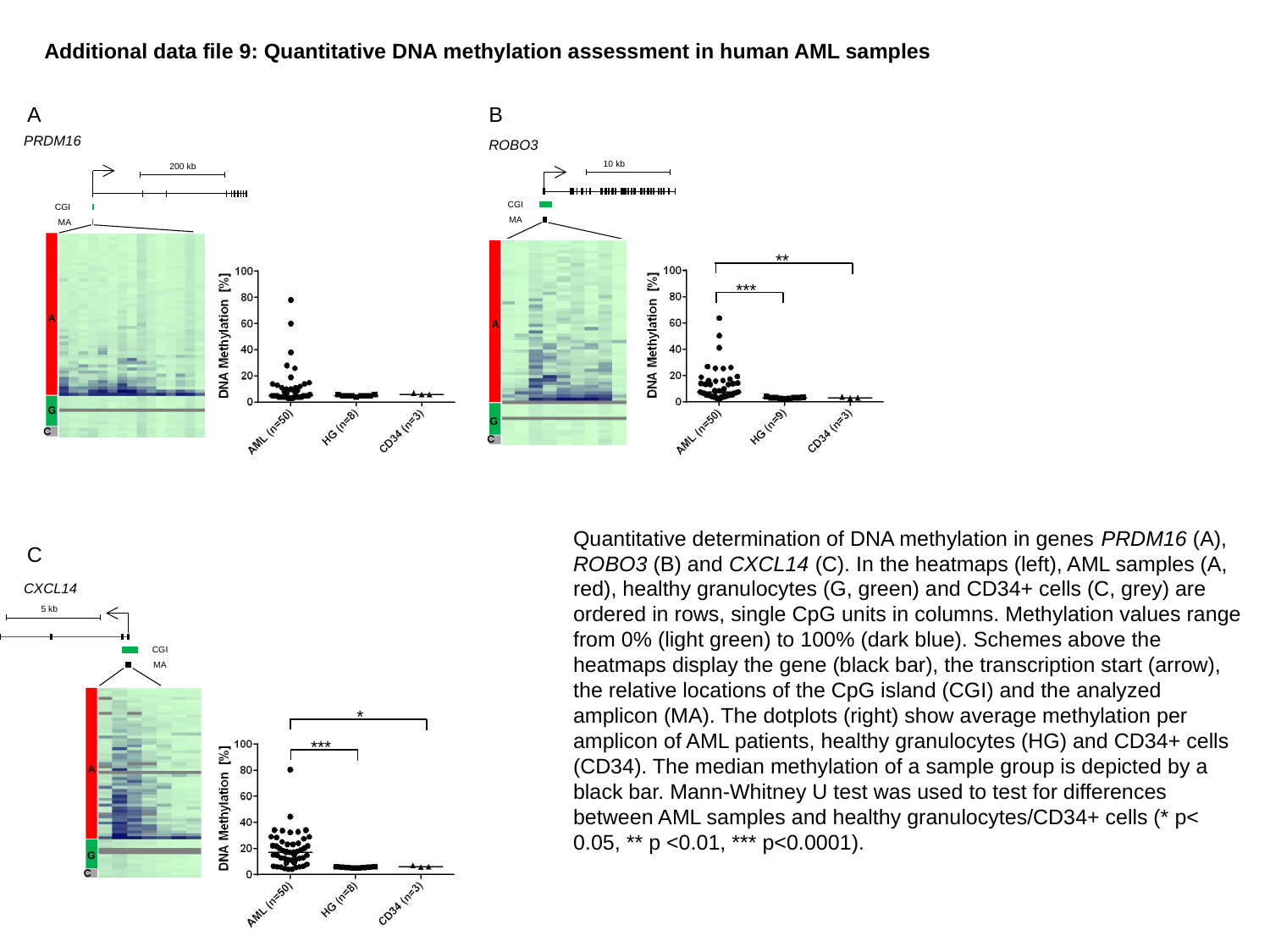

Additional data file 9: Quantitative DNA methylation assessment in human AML samples
A
B
PRDM16
ROBO3
10 kb
200 kb
CGI
CGI
MA
MA
**
***
A
A
G
G
C
C
G
Quantitative determination of DNA methylation in genes PRDM16 (A), ROBO3 (B) and CXCL14 (C). In the heatmaps (left), AML samples (A, red), healthy granulocytes (G, green) and CD34+ cells (C, grey) are ordered in rows, single CpG units in columns. Methylation values range from 0% (light green) to 100% (dark blue). Schemes above the heatmaps display the gene (black bar), the transcription start (arrow), the relative locations of the CpG island (CGI) and the analyzed amplicon (MA). The dotplots (right) show average methylation per amplicon of AML patients, healthy granulocytes (HG) and CD34+ cells (CD34). The median methylation of a sample group is depicted by a black bar. Mann-Whitney U test was used to test for differences between AML samples and healthy granulocytes/CD34+ cells (* p< 0.05, ** p <0.01, *** p<0.0001).
C
CXCL14
5 kb
CGI
MA
*
***
A
G
C
