# Supplementary material for: Early aberrant DNA methylation events in a mouse model of acute myeloid leukemia
Source: Genome Med. 2014 Apr 30;6(4):34. doi: 10.1186/gm551 (PMC4062060; doi:10.1186/gm551)
Supplement: Additional file 10 — A table showing overrepresented motifs in hypermethylated CGIs of the preleukemic stage. The table shows the results of de novo motif search of the preleukemic hypermethylated CGIs compared to whole genome background enrichment (47,511 target sequences). [file gm551-S10.docx]

**Additional data file 10: Overrepresented motifs in hypermethylated CGIs of the preleukemic stage**

| Motif | Name | P-value | log P-pvalue | q-value (Benjamini) | #Target Sequences with Motif | % of Targets Sequences with Motif | #Background Sequences with Motif | % of Background Sequences with Motif |
| --- | --- | --- | --- | --- | --- | --- | --- | --- |
| 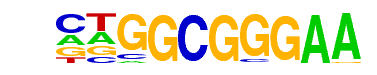 | E2F1(E2F)/Hela-E2F1-ChIP-Seq/Hoemr | 1e-2 | -5.593e+00 | 0.8009 | 89 | 11.31% | 4033 | 8.49% |
| 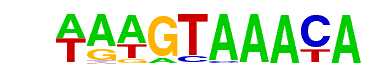 | FOXA1(Forkhead)/MCF7-FOXA1-ChIP-Seq/Homer | 1e-2 | -4.941e+00 | 0.8009 | 22 | 2.80% | 740 | 1.56% |
| 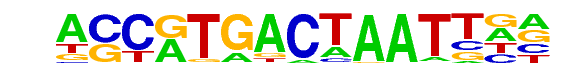 | PAX3:FKHR-fusion(Paired/Homeobox)/Rh4-PAX3:FKHR-ChIP-Seq/Homer | 1e-2 | -4.821e+00 | 0.8009 | 9 | 1.14% | 205 | 0.43% |
| 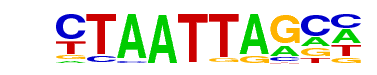 | Lhx3(Homeobox)/Forebrain-p300-ChIP-Seq/Homer | 1e-2 | -4.640e+00 | 0.8009 | 28 | 3.56% | 1041 | 2.19% |

Total Target Sequences = 787, Total Background Sequences = 47511
