# Supplementary material for: Early aberrant DNA methylation events in a mouse model of acute myeloid leukemia
Source: Genome Med. 2014 Apr 30;6(4):34. doi: 10.1186/gm551 (PMC4062060; doi:10.1186/gm551)
Supplement: Additional file 12 — A figure depicting the quantitative determination of DNA methylation in PU.1 target genes. (A-C) Heatmaps (left) and dotplots (right) of amplicons from Hes6 (A), Tal1 (B) and Itpka (C) in PU.1-wt animals and different disease stages are shown. Heatmaps display single CpG units (columns) of different PU.1-wt and PU.1-kd animals. Differently colored bars to the right of the heatmaps indicate preleukemic (P, black), early leukemic (EL, blue), late leukemic (LL, red), and PU.1-wt (W, green). In vitro methylated standard DNA (0%, 20%, 40%, 60%, 80% and 100% DNA methylation; S, grey) served as control. Methylation values range from 0% (light green) to 100% (dark blue). Schemes above the heatmaps display the gene (black bar), transcription start (arrow), the PU.1 ChIP peak (PU1), the relative location of the CpG islands (CGI) and the analyzed amplicons (MA). Dotplots show average methylation per amplicon of PU.1-wt animals and the different disease stages. Median methylation of a sample group is depicted by a black bar. Mann-Whitney U test was used to test for differences between PU.1-wt and stages and within the different stages (*P < 0.05, **P <0.01, ***P < 0.001). [file gm551-S12.pptx]

## Slide 1
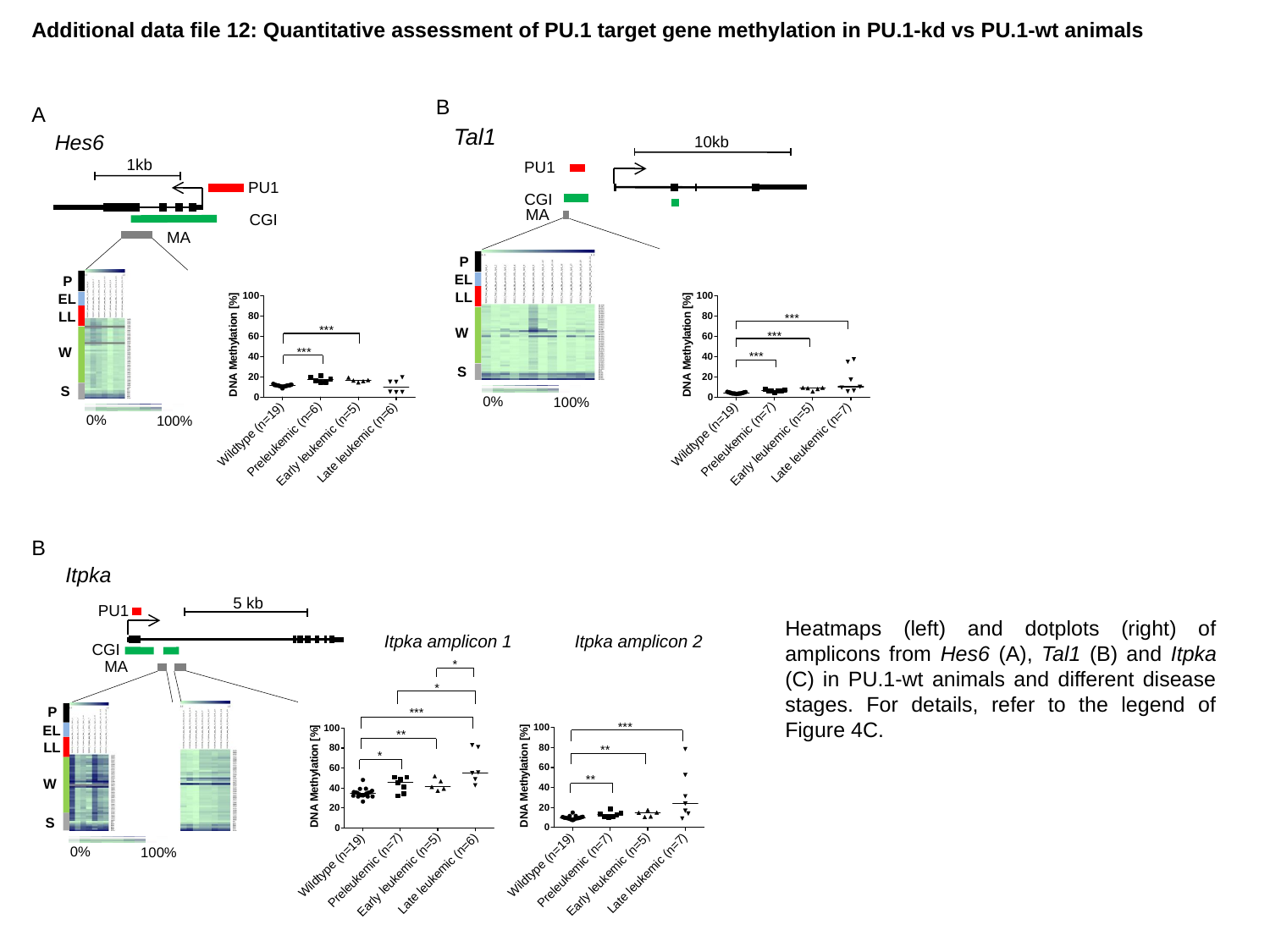

Additional data file 12: Quantitative assessment of PU.1 target gene methylation in PU.1-kd vs PU.1-wt animals
B
A
Tal1
Hes6
10kb
1kb
PU1
PU1
CGI
MA
CGI
MA
P
EL
P
LL
EL
LL
***
***
W
***
W
***
***
S
Wildtype (n=19)
Preleukemic (n=6)
Late leukemic (n=6)
Early leukemic (n=5)
Wildtype (n=19)
Preleukemic (n=7)
Late leukemic (n=7)
Early leukemic (n=5)
S
0%
100%
0%
100%
B
Itpka
5 kb
PU1
Heatmaps (left) and dotplots (right) of amplicons from Hes6 (A), Tal1 (B) and Itpka (C) in PU.1-wt animals and different disease stages. For details, refer to the legend of Figure 4C.
Itpka amplicon 2
Itpka amplicon 1
CGI
MA
*
*
P
W
S
***
***
EL
**
LL
**
*
**
Wildtype (n=19)
Preleukemic (n=7)
Late leukemic (n=7)
Early leukemic (n=5)
Wildtype (n=19)
Preleukemic (n=7)
Late leukemic (n=6)
Early leukemic (n=5)
0%
100%
